# A New Combinatorial Optimization Approach for Integrated Feature Selection Using Different Datasets: A Prostate Cancer Transcriptomic Study

**DOI:** 10.1371/journal.pone.0127702

**Published:** 2015-06-24

**Authors:** Nisha Puthiyedth, Carlos Riveros, Regina Berretta, Pablo Moscato

**Affiliations:** 1 Centre for Bioinformatics, Biomarker Discovery and Information-Based Medicine, Hunter Medical Research Institute, New Lambton Heights, NSW, Australia; 2 School of Electrical Engineering and Computer Science, The University of Newcastle, Callaghan NSW, Australia; University of Bonn, Bonn-Aachen International Center for IT, GERMANY

## Abstract

**Background:**

The joint study of multiple datasets has become a common technique for increasing statistical power in detecting biomarkers obtained from smaller studies. The approach generally followed is based on the fact that as the total number of samples increases, we expect to have greater power to detect associations of interest. This methodology has been applied to genome-wide association and transcriptomic studies due to the availability of datasets in the public domain. While this approach is well established in biostatistics, the introduction of new combinatorial optimization models to address this issue has not been explored in depth. In this study, we introduce a new model for the integration of multiple datasets and we show its application in transcriptomics.

**Methods:**

We propose a new combinatorial optimization problem that addresses the core issue of biomarker detection in integrated datasets. Optimal solutions for this model deliver a feature selection from a panel of prospective biomarkers. The model we propose is a generalised version of the *(α*,*β)-k*-Feature Set problem. We illustrate the performance of this new methodology via a challenging meta-analysis task involving six prostate cancer microarray datasets. The results are then compared to the popular RankProd meta-analysis tool and to what can be obtained by analysing the individual datasets by statistical and combinatorial methods alone.

**Results:**

Application of the integrated method resulted in a more informative signature than the rank-based meta-analysis or individual dataset results, and overcomes problems arising from real world datasets. The set of genes identified is highly significant in the context of prostate cancer. The method used does not rely on homogenisation or transformation of values to a common scale, and at the same time is able to capture markers associated with subgroups of the disease.

## Introduction

The extraction of information arising from the integration of multiple datasets and its translation into domain knowledge is a significant problem in several fields. Today, more and more biology and health related studies around the world are engaging in the useful policy of leaving their raw results available for the common good via public domain databases. This open sharing has benefitted the reproducibility of other researchers’ findings. The existing online datasets are also becoming very useful for the development of new mathematical and computational approaches for pattern recognition, machine learning and artificial intelligence methods. This healthy practice of sharing data is now being increasingly adopted by governments and scientific journals. The private and public sector is also engaged in “data-mining competitions” in which the datasets are made widely available and crowd-sourced for data analysis. In this new, digital and interconnected global research open data enterprise, this is definitely a good direction for science, research and development and we are confident to affirm that this trend is here to stay.

The term ‘meta-analysis’ generally refers to an integrated study which aims at developing a consensus of findings from individual studies. Sometimes authors use this term rather loosely meaning just a ‘review’ of a set of existing studies that are independently obtained but related to a set of common questions of interest [1]. When some conditions are met, an integrated study can help to improve the power of the analysis by increasing the total number of samples under consideration [2]. Meta-analyses are also an important tool when some of the existing studies have conflicting conclusions [3] and the overall aim is to resolve them, if possible. Increasing the detection power of smaller studies by integrating them in a larger study has also become a way to overcome research funding limitations. This is particularly the case in transcriptomics, and there is an undeniable need for new mathematical models and algorithms aimed at extracting information by jointly studying different datasets which often contain information extracted with different and ever-changing technological platforms.

The existence of large number of publicly available transcriptomic studies gives a strong motivation for the development of new mathematical methods that help to extract *panels of biomarkers* by employing several microarray datasets. In spite of the growing number of studies, an overall consensus has yet to be reached about how to do this [4, 5]. Researchers sometimes only highlight the obstacles ahead, for instance, by pointing at the essential differences in microarray platforms, experimental designs, collection procedures for samples, heterogeneities of laboratory protocols and the analysis methods used for the study [6]. Most of the studies are unable to provide a definite answer to the question of interest since too few samples are entered into the study [7]. However, all these confounding issues need to be considered and highlighting them does not diminish the need to develop integrative techniques for joint panel of biomarkers elicitation.

Many studies have shown that it is difficult to obtain a reliable result from a single dataset [8–11]. Even though some researchers may eventually procure the financial resources to conduct studies with large number of samples, leading to greater power to detect individual markers, an integrated study can provide a clearer picture as the final result would look for consensus in a number of individual studies. This shows the necessity for developing combinatorial optimization-based approaches to determine a significant list of genes from multiple platforms when we are looking at a panel that acts together for a discrimination task across several studies.

Multi-platform data integration remains challenging as the datasets from different experiments are not directly comparable due to the factors associated with the generation of the dataset [12]. Some of the challenges are simply technical in nature, for instance the genomic data may come in a wide variety of data formats, thus making direct integration difficult. The datasets can be converted to a common data format before combining them, but this is not always feasible [13]. Several methods have been proposed in the last few years for the meta-analysis of gene expression data to find the set of significant genes among the selected datasets. The existing meta-analysis methods either perform statistics for each dataset or integrate all the selected datasets into a single large dataset to estimate the differential gene expression. A rank based method proposed by Breitling *et al*. [14] and later developed by Hong *et al*. into the RankProd Bioconductor package [15], uses the fold changes between all interclass pair of samples to compute dataset ranks for each gene, then combines ranks with the geometric mean of ranks across sample pairs. MetaArray is another meta-analysis method proposed by Choi *et al*. [16] in which the data is transformed into probability of expression [17] followed by the filtering of genes based on the integrative correlation analysis. Mergemaid [18] is another package for meta-analysis that helps to integrate heterogeneous platform datasets on the basis of user-provided IDs of genes. The standardized regression coefficients and z-scores are used as a measure for the gene selection process form the integrated dataset. Although these methods are capable to select signatures from the integrated dataset of heterogeneous platforms, they are incapable to deal with genes not represented in all the datasets. A recently proposed method called NetSel [19] is a heuristic rank aggregation method for feature selection that can be applied on heterogeneous set of lists. However, RankProd is by far the most popular of those methods, and we have chosen it as a comparison benchmark.

The goal of this article is to present a new method for the integration of microarray gene expression datasets which may have been obtained using different platforms. We do this without needing to transform the values to a common uniform format and range of values. We also propose a new combinatorial optimisation approach to select the best set of common features that can discriminate the given classes. The method is a generalised version of the proven and very successful *(α*,*β)-k*-Feature Set methodology previously pioneered by our group [20, 21] and we show here how it can be applied to the combined dataset. We benchmark our new method by analysing the integration of six prostate cancer datasets produced using different platforms and highlight its main findings. We deliberately turn our attention to relatively small and also relatively old datasets, somewhat disregarded as potentially “uninteresting” due to the advances of current biotechnologies. We compare the integrated results against the collection of results of individually applying traditional statistical analysis and the *(α*,*β)-k*-Feature Set methodology to each dataset. We aim to illustrate the potential of secondary analyses of these datasets using the proposed technique.

The structure of the article is as follows; the materials and methods employed in this paper are explained in detail in Section 2; in Section 3 we present our results by applying the proposed integration and feature selection method on prostate cancer datasets. In Section 4 we present some discussion on the basis of the result. Section 5 gives a conclusion of this study and future directions.

## Materials and Methods

### 2.1 Datasets

The six publicly available prostate cancer gene expression datasets used in this study were collected from Gene Expression Omnibus (GEO) or from the original source. The details of all the datasets in this work are summarised in Table 1.

**Table 1:** Summary of datasets used in this study.

Name	Plat	Series	NS	Norm	PT	Met	Probes	EF
Singh [22]	Affymetrix [HG-U95Av2]	N/A	102	50	52	0	12558	1519
Welsh [23]	Affymetrix [HG-U95Av2]	N/A	55	9	25	21	12560	2429
Uma [24]	Affymetrix [HG-U95B]	E-GEOD-6919	80	17	63	0	37691	3484
L-2695 [25]	SHBB	GSE3933	26	9	13	4	44161	4288
L-3044 [25]	SHCQ	GSE3933	41	16	23	2	43009	4082
L-3289 [25]	SHBW	GSE3933	45	16	26	3	43009	4953

**Name** is the name assigned to the study throughout this paper. **Plat** is the platform details of each dataset. **Series** is the Gene Expression Omnibus Series identifier for the dataset. **NS** is the original number of samples in the study, of which **Norm** are the number of healthy tissue samples, **PT** are the number of primary tumour samples, **Met** is the number of metastasis samples present in each dataset, **Probes** is the number of probes present in each dataset, **EF** is the number of probes present after entropy filtering.

The selected datasets have been generated using two different platforms. The gene expression levels of three of them were measured using cDNA two-channel arrays and the other three using Affymetrix arrays. The datasets are named according to the name of the first author of the published article. As shown in, the last three datasets are collected form the same article, so the datasets have been named with the first author’s initial and the GEO platform number (eg. L-2695). Details of the datasets are as follow.

In [22], Singh et al. introduced an outcome prediction model to distinguish between tumour and normal samples. The dataset used in this study contains 102 tissue samples collected after radical prostatectomy. The sample consists of 50 normal samples and 52 primary prostate cancer samples. This dataset was generated using Affymetrix HG-U95A v2 (GPL8300) arrays.

The second dataset has been contributed by Welsh et al. [23] in 2001. The study investigates a therapeutic approach to differentiate the tumour and normal samples. The dataset contains 55 samples that are hybridised to HG-U95A v2 (GPL8300) arrays. The samples are of 25 primary tumour and 9 normal tissues and the rest of the samples were taken from different donors with different types of cancers.

The third dataset has been published by Uma et al. in 2007 [24]. This study introduces an experimental design to address the differences in cellular content between primary and metastatic tumours. The dataset contains 63 tumour tissue samples and 17 normal tissue samples and has been produced using Affymetrix HGU95Av2 arrays.

Lapointe et al. [25] introduced a hierarchical clustering technique to distinguish tumour from normal samples and to identify the subclasses of prostate cancer in 2004. This study was performed using three different datasets produced using cDNA two-channel arrays; the first Lapointe dataset (L-2695) contains 26 samples (13 primary tumour tissue, 9 normal tissue and 4 metastasis tissue samples). The second Lapointe dataset (L-3044), with a total sample count of 41, has 23 primary tumour samples, 16 normal samples and 2 metastasis samples. The third dataset (L-3289) contains a total of 45 samples, of which 26 are primary tumour, 16 normal and 3 metastasis samples.

We have restricted our study only to those samples which originate in either primary tumours or normal tissue. The total numbers of samples are then 319, of which 202 are primary tumours and the rest are from normal tissue.

### 2.2 Integration method

The direct integration of microarray gene expression data from multiple platforms is, in principle, greatly facilitated when there exists commonality between the platforms used. However different gene expression platforms will target genes or transcripts differently by using different sets of probes. There may be many probes mapping the same gene due to duplicate spotted probes in microarray chips. On the other hand, there may be a single probe that maps to several genes (or loci) if the specificity of the probe sequence is not good enough. These probes must be discarded from the preliminary analysis as it is difficult to analyse these multiple genes. In addition, the interpretation of the results via Gene Ontology or pathway-informed databases could be compromised by the multiple mapping problems. In addition to these difficulties, we may also face the problem that one probe targeting different regions of the same gene could be indirectly monitoring possible different abundances of protein isoforms. This many-to-many nature of the mapping problem makes it difficult to take a simplistic approach to the essentially different maps that platforms produce by their probe sets.

In this contribution, we map at the gene level. In order to map the probes across the platforms in Table 1 to genes, we have used a simple alignment policy, explained below; with no distinction of isoforms and also ignored the mentioned problems. The probes were mapped using the hg19-GRCh37 version of the Genome Browser’s table produced by the Genome Reference Consortium to avoid the misnaming and misalignment of genes. In order to obtain a relatively large number of probes that could be used in the final integrated dataset, we collected those that satisfy any of the given three conditions:

- Where the probes are targeting the same sequence
- Where the targeting sequences are overlapping
- Where the targeting sequences are at a distance of at most 1000 base pairs

The probes from each dataset have been mapped to genes and the associated transcription start and end position of the targeting genes compared according to the conditions mentioned above. Whenever there is a common targeting gene for different probes from multiple datasets, we consider the different combinations of those probes in the combined dataset. Similarly, if the features (the transcription start and end sequences) have an overlap between them, or are at a distance of at most 1000bp, the combination of those probes is also selected to be part of the combined dataset. The selected list of combination of probes is given in the Supplementary Materials (S1 Table). Each unique combination of probes from different datasets becomes a feature in the combined dataset.

### 2.3 Feature selection method

Initially, we used Fayyad and Irani’s entropy-based heuristic on each individual dataset to remove uninformative features. This univariate selection mechanism is a pre-processing step related to the Minimum Description Length Principle (MDL) [26]. The purpose of using this step in this method is twofold: it removes features that are not significantly different in healthy and disease samples (thus it helps by reducing the dimensionality of the problem), and second it helps discretise the values (which in turn facilitate the combinatorial approach).

In this contribution we propose and analyse a new combinatorial approach to select a set of *k* significant features that can explain the multi-platform integrated datasets. We call this problem the Coloured *(α*,*β)-k*-Feature Set problem. The approach is a generalised version of the *(α*,*β)-k*-Feature Set problem methodology [27, 28] which is a supervised feature selection method to select a significant set of features that can collectively separate the sample groups. The method has been successfully used in several studies by Moscato et al. for finding biomarkers for different diseases [20, 21, 28–34].

The *(α*,*β)-k*-Feature Set problem provides a significant set of genes that collectively maximise the inter-class discrimination and the intra-class coherency [33]. The method seeks to differentiate all sample pairs which belong to different classes by selecting a minimum set of genes that do not necessarily present a uniform expression level across samples in each class but collectively provide the maximum amount of evidence. In contrast, rank methods that score and order genes by their differential expression across the classes bring gene sets that may not work together as a signature, particularly in complex diseases whose molecular characterisation may present subgroups.

The mentioned feature selection method works well with a single uniform dataset, but not for an integrated dataset. The Coloured *(α*,*β)-k-*Feature Set problem handles the integrated dataset in a consistent manner and selects features that differentiate sample pairs across the datasets. The application of an *(α*,*β)-k-*Feature Set problem based method for meta-analysis thus helps provide the best set of features from the combined dataset, allowing researchers to reveal the genetic pathways that take part in the development of the disease.

Here we more formally present the decision versions of the generalization of the *k*-Feature Set problem called the *(α*,*β)-k*-Feature Set problem, the Coloured *(α*,*β)-k*-Feature Set problem and the Generalised *(α*,*β)-k*-Feature Set problem. In what follows, let B represent the set of binary values, i.e. B={0,1}; let *n* be the number of features and *m* the number of samples, *p* be the number of sample groups (i.e., different platforms/cohorts/datasets) and the tuple *y* be the class labels of the samples.

### 2.3.1 (α,β)-k-Feature Set.

#### Instance:

A set $X=\{x_{i}|x_{i}\in B^{n}\wedge 1\leq i\leq m\}$, a tuple *y* ∈ *B* ^*m*^, integers *α* > 0, *β* ≥ 0, *k* > 0

#### Parameters:

*α*, *β* and *k*

#### Question:

Is there a set *I* ⊆ {1,…, *n*} with |*I*| ≤ *k* such that for all *i*, *j* ∈ {1,…, *m*}

- If *y*_*i*_ ≠ *y*_*j*_ there exists $I_{i,j}^{\alpha }\subseteq I$ with $\left|I_{i,j}^{\alpha }\right|\geq \alpha$ such that *x*_*i*,*s*_ ≠ *x*_*j*,*s*_ for all $s\in I_{\left(i,j\right)}^{\alpha },$
- If *y*_*i*_ = *y*_*j*_ there exists $I_{i,j}^{\beta }\subseteq I$ with $|I_{\left(i,j\right)}^{\beta }|\geq \beta$ such that *x*_*i*,*s*_ = *x*_*j*,*s*_ for all $s\in I_{\left(i,j\right)}^{\beta }?$

Detailed explanation of safe reduction rules that help to reduce the dimensionality of the *(α*,*β)-k* Feature Set problem are given in [20, 32].

### 2.3.2 Coloured *(α*,*β)-k-*Feature Set.

#### Instance:

A set $X=\{x_{i}|x_{i}\in B^{n}\wedge 1\leq i\leq m\}$, a colouring function *c*: {1,…, *m*} → {1,…, *p*}, a tuple $y\in B^{m}$, integers *α* > 0, *β* ≥ 0, *k* > 0

#### Parameters:

*α*, *β* and *k*

#### Question:

Is there a set *I* ⊆ {1,…, *n*} with |*I*| ≤ *k* such that for all *i*, *j* ∈ {1,…, *m*} where *c*(*i*) = *c*(*j*)

- If *y*_*i*_ ≠ *y*_*j*_ there exists $I_{i,j}^{\alpha }\subseteq I$ with $\left|I_{i,j}^{\alpha }\right|\geq \alpha$ such that *x*_*i*,*s*_ ≠ *x*_*j*,*s*_ for all $s\in I_{\left(i,j\right)}^{\alpha },$
- If *y*_*i*_ = *y*_*j*_ there exists $I_{i,j}^{\beta }\subseteq I$ with $|I_{\left(i,j\right)}^{\beta }|\geq \beta$ such that *x*_*i*,*s*_ = *x*_*j*,*s*_ for all $s\in I_{\left(i,j\right)}^{\beta }?$

In words, the Coloured *(α*,*β)-k*-Feature Set problem instance is constructed from a collection of individual *(α*,*β)-k*-Feature Set instances with common features, where the comparison of feature values is limited to sample pairs formed from each individual instance. The “coloured” name stems from assuming samples in each individual instance are coloured with the same unique colour, then only same coloured samples can be combined in pairs.

It is evident that the same set of data reduction rules presented in [21] for the *(α*,*β)-k*-Feature Set problem applies to an instance of the Coloured *(α*,*β)-k*-Feature Set problem, as the latter is formally equivalent to a larger instance of an *(α*,*β)-k*-Feature Set problem by an appropriate relabelling of samples.

### 2.3.3 Generalised *(α*,*β)-k-*Feature Set.

In the most general form appropriate for meta-analysis of datasets with common features, the *(α*,*β)-k*-Feature Set problem can be stated as follows:

#### Instance:

A set $X=\{x_{i}|x_{i}\in B^{n}\wedge 1\leq i\leq m\}$, a function g:{1,…,m}×{1,…,m}→B, a tuple $y\in B^{m}$, integers *α* > 0, *β* ≥ 0, *k* > 0

#### Parameters:

*α*, *β* and *k*

#### Question:

Is there a set *I* ⊆ {1,…, *n*} with |*I*| ≤ *k* such that for all *i*, *j* ∈ {1,…, *m*} where *g*(*i*, *j*) = 1

- If *y*_*i*_ ≠ *y*_*j*_ there exists $I_{i,j}^{\alpha }\subseteq I$ with $\left|I_{i,j}^{\alpha }\right|\geq \alpha$ such that *x*_*i*,*s*_ ≠ *x*_*j*,*s*_ for all $s\in I_{\left(i,j\right)}^{\alpha },$
- If *y*_*i*_ = *y*_*j*_ there exists $I_{i,j}^{\beta }\subseteq I$ with $|I_{\left(i,j\right)}^{\beta }|\geq \beta$ such that *x*_*i*,*s*_ = *x*_*j*,*s*_ for all $s\in I_{\left(i,j\right)}^{\beta }?$

The Generalised *(α*,*β)-k*-Feature Set problem has been devised to deal with the more general situation in which some samples in one sample group may be compared to samples in another sample group, for example. The binary function *g*(*i*, *j*) indicates when feature values for a given arbitrary sample pair (*i*, *j*) can be compared.

In all previous formulations, the samples have been presented as an array of *n*+1 binary values, although this is not strictly necessary. The class label can be a categorical variable taking values over a (typically small) set of categories or classes. The features can have values of any kind, as long as there exists a meaningful comparison able to decide if any two values are considered equal or different.

### 2.3.4 Coloured *(α*,*β)-k-*Feature Set as an Integer Programming Problem.

Next, we present the Coloured *(α*,*β)-k-*Feature Set problem as an Integer Programming optimisation problem. Let *p*, *n*, *m* and *y* be as given before. As the sample groups are disjoint, there are no common samples between any two of them. For any sample *j* and any feature *s* ∈ {1,…, *n*}, let *c*_*j*_ ∈ {1,…, *p*} be the sample group it belongs to, and *x*_*js*_ the value of the feature for the sample. For any sample pair (*i*, *j*) let

$$a_{ijs}=\{\begin{array}{l} 1\;if\;y_{i}\neq y_{j}\;and\;c_{i}=c_{j}\;and\;x_{is}\neq x_{js}\\ 0\;otherwise \end{array}$$

and

$$b_{ijs}=\{\begin{array}{l} 1\;if\;y_{i}=y_{j}\;and\;c_{i}=c_{j}\;and\;x_{is}=x_{js}\\ 0\;otherwise \end{array}$$

The objective function and constraints for the Coloured *(α*,*β)-k*-Feature Set problem integer programming optimisation models are given below, where the binary variable *f*_*s*_ is 1 if the feature *s* is selected to the feature set, and 0 otherwise. The problem seeks the minimum of:

$$k=min\sum_{s=1}^{n}f_{s}$$ (1)

subject to the conditions:

$$\sum_{s=1}^{n}a_{ijs}f_{s}\geq \alpha \;\forall \;\left(i,j\right)$$ (2)

$$\sum_{s=1}^{n}b_{ijs}f_{s}\geq \beta \;\forall \;\left(i,j\right),$$ (3)

where:

$$f_{s}\in \left\{0,1\right\}$$

A Coloured *(α*,*β)-k*-Feature Set problem instance can have more than one optimal solution with k features in each. This multiplicity is resolved by a subsequent optimisation problem which searches for the solution of size k with maximum cover. We then define the optimal solution of the Coloured *(α*,*β)-k*-Feature Set problem as the one that maximises:

$$V=max\sum_{s=1}^{n}e_{s}f_{s}$$ (4)

subject to the conditions:

$$\sum_{s=1}^{n}f_{s}=k$$ (5)

$$\sum_{s=1}^{n}a_{ijs}f_{s}\geq \alpha \;\forall \;\left(i,j\right)$$ (6)

$$\sum_{s=1}^{n}b_{ijs}f_{s}\geq \beta \;\forall \;\left(i,j\right),$$ (7)

where:

$$f_{s}\in \left\{0,1\right\}$$

In Eq 4, the cover *e*_*s*_ is the number of pairs of samples that feature *s* covers, and can be specified as:

$$e_{s}=\sum_{i,j\in \left\{1,\ldots ,m\right\}}\left(a_{ijs}+b_{ijs}\right)$$

The solution of the optimisation problem (1–3) requires the specification of the parameters *α* and *β*. One way of requiring a robust solution of the problem is to specify *α* as large as possible. This value is determined by the instance of the problem, and is equal to the minimum number of features that differentiate any sample pair of different class labels. Once the value of *k* is obtained with *β* = 0, we can then repeatedly solve the problem (4–7) for increasingly large values of *β* in (7), until the problem becomes unfeasible. The last feasible solution is the signature sought.

A final note about the computational complexity of this family of problems. The *(α*,*β)-k*-Feature Set problem is at least as complex as the classical *k*-Feature Set problem, which is NP-complete [35, 36]. The *(α*,*β)-k*-Feature Set problem is not only NP-complete, but W[2]-complete [37, 38].

### 2.4 t-test

In order to benchmark against traditional statistical methods, we perform a t-test analysis of the individual datasets. The t-test is a statistical significance test method used here to select genes that exhibit differential gene expression between two different conditions [39], in our case normal vs. primary tumour, above a certain *p*-value level of confidence. The procedure of *t*-test is described below:

Let *S*_1_ and *S*_2_ be the mean values of a particular gene in the two different class labels 1 and 2, of sizes *m*_1_ and *m*_2_. The *t*-statistic for this particular gene is computed as:

$$t=\frac{S_{1}-S_{2}}{X\sqrt{\frac{1}{m_{1}}+\frac{1}{m_{2}}}}$$

where *X* is the pooled sample variance

$$X=\sqrt{\frac{m_{1}x_{1}^{2}+m_{1}x_{2}^{2}}{m_{1}+m_{2}-2}}$$

Here $x_{1}^{2}$ and $x_{2}^{2}$ are the variance of replicated observations in each condition and *n*_1_ + *n*_2_ − 2 is the number of degrees of freedom. In our study we used the ‘genefilter’ Bioconductor package [40] with a chosen *p*-value of 10^−4^ to perform our *t*-test.

### 2.5 RankProd

We compare our results to those obtained by another popular meta-analysis method. RankProd is a non-parametric meta-analysis tool introduced by Hong et al. [15] to detect differentially expressed genes. It arguably is the most widely used gene expression meta-analysis method, and is provided as a Bioconductor package that modifies and extends the rank product method proposed by Breitling et al. [14]. Fold Change (FC) is used as scoring criteria to rank and compare genes within each dataset. An overall ranked gene list is produced by aggregating the individual ranks across datasets.

A pair-wise fold change (*p*FC) is computed for each gene *g* within a given dataset *k* as,

$$T_{1}^{g}/C_{1}^{g},T_{1}^{g}/C_{2}^{g},\ldots ,T_{2}^{g}/C_{1}^{g},\ldots ,T_{n_{Tk}}^{g}/C_{n_{Ck}}^{g}$$

in which $T_{j}^{g}$ and $C_{l}^{g}$ are the expression values of gene *g* for sample *j* (belonging to experimental condition *T–*e.g. “tumour”) and *l* (belonging to experimental condition *C–*e.g. “control”), and $n_{T_{k}}$ and $n_{C_{k}}$ are the number of replicates which produce a total of $K_{k}=n_{T_{k}}\times n_{C_{k}}$ *p*FC values per gene. Then the corresponding *p*FC ratios are ranked and are denoted as *r*_*gi*_, where *g* = 1,…, *G* represents the number of genes and *i* = 1,…, *K*_*k*_ represents the pairwise comparison between samples. The rank product of each gene *g* is defined as the geometric mean,

$$RP_{g}={\left(\prod_{i}^{K}\;r_{gi}\right)}^{1/K}$$

Expression values for each gene within each datasets is independently permuted *L* times and produce $RP_{g}^{\;(l)}$ where *l* = 1,…, *L* by repeating the above steps. A reference distribution is obtained from all $RP_{g}^{\;(l)}$ and the adjusted p-value and the false discovery rate for each gene calculated.

In this study, the datasets are combined in terms of common genes across the platforms. We have applied RankProd on the combined dataset to select genes associated to the condition being investigated.

### 2.6 Robustness

To evaluate the robustness of our method with respect to perturbations in the data we have performed a series of experiments. The presence of noise in the gene expression data is difficult to estimate, as it depends on platform-specific factors as well as experimental conditions. However, the final manifestation of perturbations in the datasets would be a change in the composition of the set of probes that pass the MDL criterion. We have thus analysed the robustness of the final integration results with respect to varying compositions of the individual datasets, for different perturbation models, inspired by the ‘leave one out’ approach. Specifically, we have modelled the following setups: a) removal of one, two and five genes from the combined dataset, and b) removal of one gene from one and two individual datasets. In order to estimate the worst case scenario, all genes were restricted to those that appear in our final signature as expressed in all six datasets. In each case, all combined probes corresponding to the chosen gene(s) are removed. An integrated signature is then obtained and compared with our original signature. The procedure is repeated 10 times for the a) case and 5 times for the b) case, with random selection of gene(s) and dataset(s), and average results reported.

## Results

As we have mentioned before, to evaluate the applicability and usefulness of the proposed method, we have selected primary tumours and normal samples from six prostate cancer datasets measured with different platforms. Since the method proposed in this work is a generalization of the *(α*,*β)-k-*Feature Set approach for probe set selection, the most natural comparison is to evaluate their results by contrasting them to those that are obtained by applying *(α*,*β)-k-*Feature Set individually. This means that we need to solve the feature set problem for each of the datasets and find the individual gene signatures that discriminate the sample classes. We then apply our proposed method, Coloured *(α*,*β)-k-*Feature Set problem. This experimental scenario has been designed to observe the benefit of an integrated approach against the comparison of gene lists obtained by analysing each of the individual experiments by separate.

For completeness we include the results of the individual datasets *t*-test analysis. In this way, we compare the benefits of our integrated approach against a frequently used feature selection method based on a univariate statistical test.

To evaluate the relative performance of the proposed method, we compare results obtained by the RankProd method. As explained in the introduction, many meta-analysis methods are not applicable in the general conditions of a multi-platform meta-analysis. RankProd is a popular choice that is able to do it, and somewhat similar in the sense that ranks genes based on the comparison of values for pairs of samples of different class labels.

### 3.1 Individual *(α*,*β)-k-*Feature Set problem results

The application of the *(α*,*β)-k*-Feature Set methodology consists of a pre-filtering step and the solution of a combinatorial optimisation problem. The pre-filtering selects features based on the class information content and discard less informative features thus reducing the dimensionality for the subsequent combinatorial problem. Details of the methods are provided in Section 0. The characteristics of the individual dataset result are given in Table 2.

**Table 2:** The results of the numerical solution of the *(α*,*β)-k-*Feature Set problem on each of the six individual datasets.

Dataset	Feat.No	After EF	*α*	*β*	*k* (signature size)
Singh	12558	1519	215	329	754
Welsh	12560	2429	1188	1068	1768
Uma	37691	3484	881	1079	1857
L-2695	44161	4288	2266	2421	3533
L-3044	43009	4028	966	862	1800
L-3289	43009	4953	1397	1216	2696

**Dataset** is the short name used in this paper for the dataset. **Feat. No** is the initial number of features (probes) present in the dataset, **After EF** is the number of features after applying entropy filtering, ***α*** and ***β*** are the values for the parameters *α* and *β* for any feasible solution, and ***k* (signature size)** is the number of probes in the resulting solution to the individual *(α*,*β)-k-*Feature Selection problem for the dataset. For method details refer to Materials and Methods.

Each dataset resulted in molecular signatures with a large number of genes (provided in the S2 Table). Surprisingly the number of common genes between them is only seven and they represent a negligible overlap of results between all experiments. This shows the need of an integrative method as it would be infeasible to come up with any form of statistical support that could link these genes to putative pathways that could be deregulated. On the positive side, however, all seven genes in the overlap already have reported association with prostate cancer. The list and literature references are given in Table 3.

**Table 3:** List of common genes among all the individual dataset results from Table 2.

Gene Symbol	Gene Name	Reference
EEF2	Eukaryotic Translation Elongation Factor 2	[82, 83]
SPG20	Spastic Paraplegia 20	No associated reference
ERG	Erythroblastosis Virus E26 Oncogene Homolog	[89, 90]
AMACR	Alpha-Methylacyl-CoA Racemase	[59, 91]
SOX4	SRY (Sex determining Region Y)-box 4	[64, 90]
APOC1	Apolipoprotein C-I	[92, 93]
GUCY1A3	Guanylate Cyclase 1, soluble, alpha 3	[94]

**Gene Symbol** is the official gene symbols. **Gene Name** is the expanded gene name. **Reference** is the reference for each gene which shows the relation with prostate cancer.

### 3.2 *t*-test Results

In order to have a baseline for comparison with another methodology common in practice, a *t*-test was conducted on each of the six individual datasets to compare the gene expression levels of normal and primary tumour samples. We compare this with our individual *(α*,*β)-k-* Feature Selection results. The method is explained in detail in Section 2 and the individual dataset results are given in Table 4.

**Table 4:** *t*-test results on individual dataset.

Dataset	Feat.No	Signature size
Singh	1519	616
Welsh	2429	717
Uma	3484	690
L-2695	4288	286
L-3044	4028	654
L-3289	4953	647

**Dataset** is the short name used in this paper for the dataset. **Feat.No** is the number of features (probes) present in the dataset before applying t-test, and **Signature size** is the number of genes in the resulting solution for each dataset. For method details refer to Section 2.

Large numbers of genes are filtered out from each dataset using the *t*-test approach (the resulted list of genes is provided in S3 Table). The only genes common to the results of all six experiments are EPCAM (epithelial cell adhesion molecule), also a known marker [17, 41–48], SOX4, EEF2 and AMACR. This shows even less genes in common than the overlap between individual *(α*,*β)–k-*Feature Selection signatures. The *t*-test result is consistent with the individual *(α*,*β)-k-*Feature Selection results as SOX4, EEF2 and AMACR are also present in the overlapping genes of individual *(α*,*β)-k-*Feature Selection results.

### 3.3 Coloured *(α*,*β)-k*-Feature Set problem results

To apply the proposed feature selection method we prepared a combined dataset collecting the entropy filtered probes from the individual studies. This ensures that selected probes in each individual study carry some differential expression information with respect to the sample classes, and also provides a well-defined discretization which respects the individual study conditions.

Probes in one platform were matched to probes in another platform based on gene names and genomic positions as explained in Section 0. The combined dataset contains 319 samples and 16157 combined probes. Out of these, 1405 contain values for all six datasets and 10729 for three or more datasets which is annotated to 1454 unique genes. The number of combined probes covering only one dataset was 3425. This uneven cover of datasets is due to some probes being discarded by the entropy filtering as uninformative only in some datasets and not in others. However, the large number of combined probes with values in three or more datasets indicates a good level of coverage after dataset integration.

We then applied Coloured *(α*,*β)-k-*Feature Set selection methodology in the combined dataset and obtained a resulting list of 3190 combined probes with a maximum of α and β value of 612 and 776 respectively, which corresponded to 1788 unique genes. The resulted number of probes for selected number of datasets and their corresponding number of genes are given in Table 5 (The list of genes can be found in S4 Table).

**Table 5:** Result of Coloured *(α*,*β)-k-*Feature Set selection methodology.

No of Datasets	No of Combined Probes	No of Genes
Four or more	2272	327
Five or more	1806	186
Six	792	120

**No of Datasets** is the considered number of datasets to find the coverage. **No of Combined Probes** is the resulted number of features after applying Coloured *(α*,*β)-k*-Feature Set selection methodology and **No of Genes** is the number of genes corresponds to the number of combined probes.

**Fig 1:**
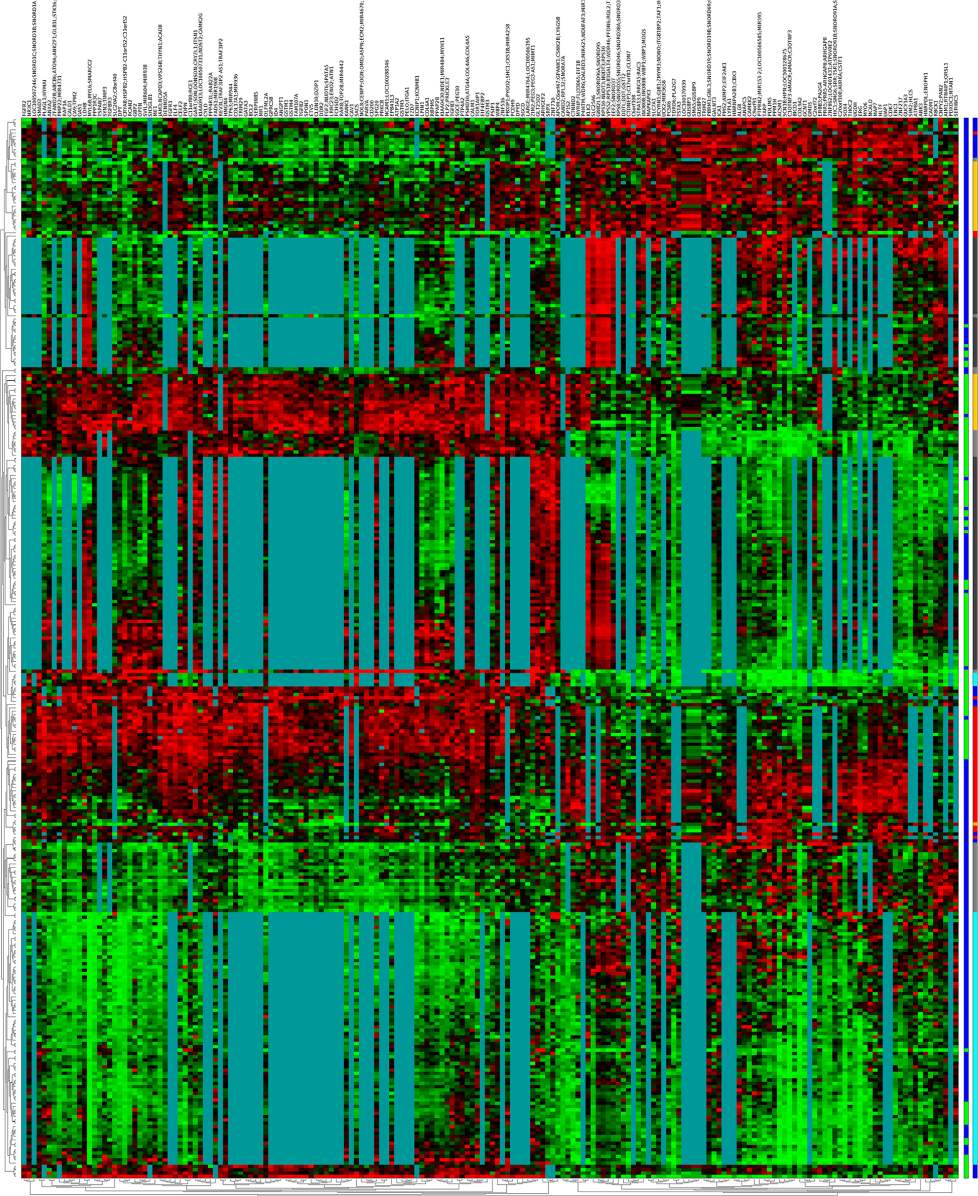
Heatmap for the Coloured *(α*,*β)-k*-Feature Selection resulted genes that cover five or more datasets. It contains 186 up and down regulated genes (columns). The genes are ordered using a memetic algorithm introduced by Moscato et al. in [49]. The blocks of greenish blue colour represent the absence of gene values in particular datasets. The first colour bar at the right indicates Primary Tumour (blue) and Normal [13] samples. The second colour bar represents each sample group in different colour. L-2695 (blue), L-3044 (red), L-3289 (orange), Welsh (grey), Uma (cyan) and Singh (dark grey).

**Fig 2:**
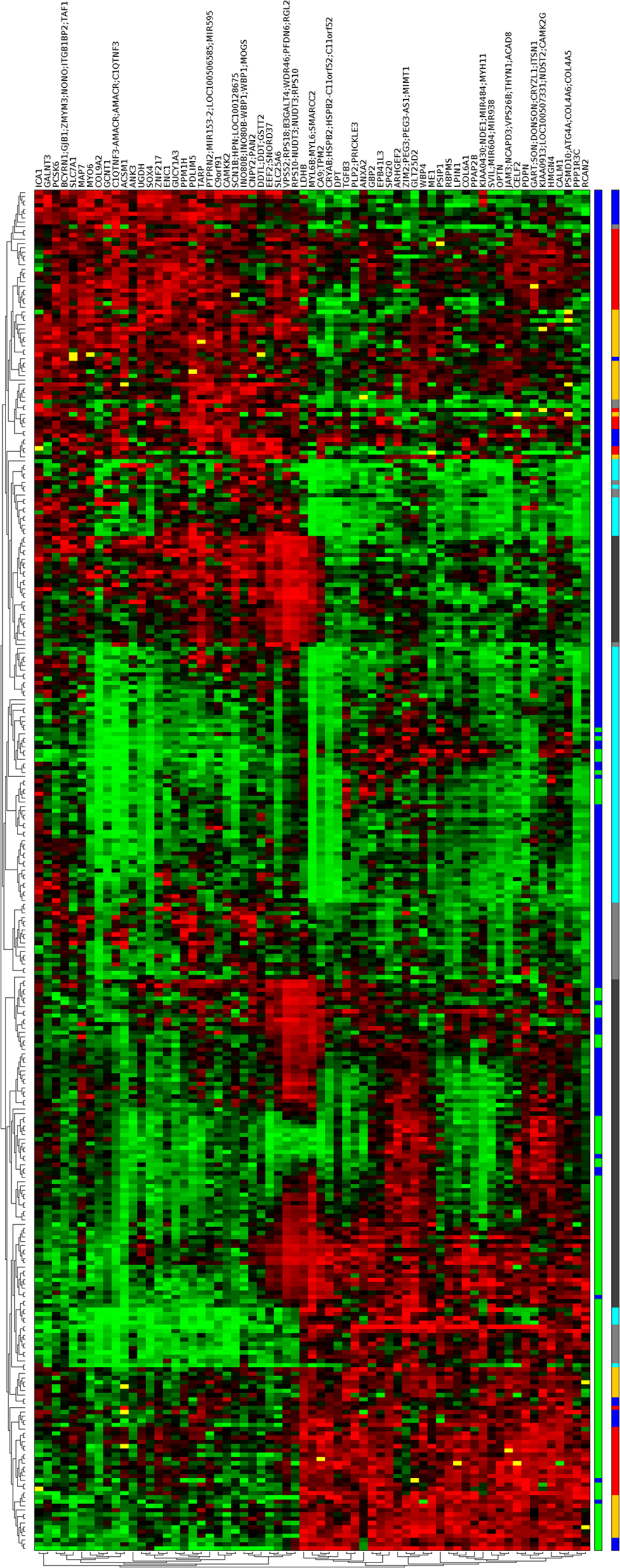
Heatmap for the Coloured *(α*,*β)-k*-Feature Selection resulted genes that cover six datasets. There are 120 up and down regulated genes (columns) which are differentially expressed between normal and tumour classes. The two colour bars at the right represent the ordering of samples and sample groups, respectively, as explained in Fig 1.

An gene ordering algorithm, presented in [49], has been applied on this set of genes to generate a heatmap that brings out the correlation between the resulted genes and is shown in Fig 1, heatmap for the 186 genes that cover five or more datasets and Fig 2 for the 120 genes that cover all six datasets, respectively.

If we consider the genes that appear in the overlap of the *t*-test, *(α*,*β)-k-*Feature Selection and Coloured *(α*,*β)-k-*Feature Selection results individually, we get very few genes in the case of *t*-test and the individual *(α*,*β)-k-*Feature Selection, but Coloured *(α*,*β)-k-*Feature Selection gives 120 unique genes (The list of genes and the details can be found in S5 Table). That shows a significant difference in the number of common genes from 7 to 120. The number of overlapping genes in different method is given in Table 6.

**Table 6:** Overlapping genes in t-test, Coloured *(α*,*β)-k* and *(α*,*β)-k*-Feature Selection.

Number of Datasets	*t*-test	*(α*,*β)-k-*Feature Selection	Coloured *(α*,*β)-k-*Feature Selection
Six	4	7	120
Five or more	22	57	327
Four or more	36	139	623

**Number of Datasets** shows the number of datasets considered to find the overlapping. ***t*-test** gives the number of overlapping genes in t-test results for the considered datasets, ***(α*,*β)-k* Feature Selection** gives the number of overlapping genes between individual *(α*,*β)-k* feature selection result for each case. **Coloured *(α*,*β)-k*-Feature Selection** gives the number of common genes in the result of Coloured *(α*,*β)-k*-feature selection considered case of datasets. For method details refer to Section 2.

### 3.4 RankProd Result

The RankProd ordered the genes by increasing pfp (percentage of false positive likelihood) value, and the top genes with a 0.05 pfp cut-off from both up and down regulated list of genes were used for the comparison. This resulted in a list of 1883 genes from the combined dataset (the list of genes can be found in S6 Table).

The comparison between Coloured *(α*,*β)-k-*Feature Set methodology result (120 genes) with the RankProd result shows that 80 out of our 120 genes are present in the top listed genes of RankProd result of the combined dataset. This signals a high level of agreement between the two meta-analysis methods. The comparison of Coloured *(α*,*β)-k-*Feature Set problem result and RankProd result is given in Table 7. All genes in RankProd result also appearing in Coloured *(α*,*β)-k-*Feature Set result are marked in a supplementary material table (S6 Table). In addition to the common 80 genes, notice there are also genes marked as four or five datasets in Coloured *(α*,*β)-k* because they have been filtered by the entropy filtering as non-informative for one or two datasets. This increases the agreement to 260 genes out of 327 (almost 80%) appearing in four or more datasets for Coloured *(α*,*β)-k*.

**Table 7:** Comparison of Coloured *(α*,*β)-k-*Feature Set problem result and RankProd result.

	RankProd			No of CABK resulted genes		
Dataset	No of genes as input	pfp Cut off	No of resulted genes	Six datasets (120)	Five datasets (327)	Four datasets(623)
Combined dataset	6929	0.05	1883	80	169	260
	6929	0.01	1484	58	140	214

**RankProd** is the result of RankProd for Combined dataset with 0.05 and 0.01 pfp (percentage of false positive likelihood cut-off). **No of CABK resulted genes** is the number of genes resulted from Coloured *(α*,*β)-k-*Feature Set problem which covered six, five and more, four and more datasets.

However, further analysis with RankProd including genes missing in one or more datasets places these genes at the top of the list, making further analysis difficult. Similarly, when genes with sparse missing values are included, these genes artificially escalate in the ranked lists towards the significant side as more missing values are introduced. This shows the inability of this method to deal with two frequent situations found in microarray datasets.

### 3.5 Robustness

To evaluate the robustness of the proposed method, we performed a sensitivity analysis by iteratively removing one or a set a genes at a time and compared the result with the original result. Summary results are given in Table 8. On average, our results remain the same for more than 97% of the signature list, while the signatures size remain essentially the same (less than 0.5% increase in the worst case). This point to a highly robust result which does not depend on a (small) set of genes, even if they are on the high coverage set.

**Table 8:** Result of sensitivity analysis.

	Case a			Case b	
	Exp-1 (1 gene)	Exp-2 (2 genes)	Exp-3 (5 genes)	Exp-4 (1 gene / 1DS)	Exp-5 (1 gene / 2DS)
Average Signature Length	3203.1 (28.68)	3201.4 (28.21)	3204.9 (9.48)	3190.2 (0.45)	3190.8(1.30)
Average % Overlap with Original	97.42 (3.31)	97.62 (3.03)	98.04 (0.51)	99.41 (0.52)	99.15 (0.63)
Average Number of New Features	46.1 (11.47)	50.5 (17.67)	77.3 (17.97)	18.6 (16.80)	27.6 (20.98)
Average Cover of New Features	3.6	3.17	3.4	1.47	1.4
Average Signature length variation	0.41%	0.36%	0.47%	0.01%	0.03%

**Case a** is the result of sensitivity analysis after removing one gene (**Exp-1**), two genes (**Exp-2**) and five genes (**Exp-3**) from the combined dataset. **Case b** gives the result of sensitivity analysis after removing one gene from one (**Exp-4**) and two (**Exp-5**) individual datasets. For more details refer to Section 2. Values in parenthesis are the standard deviations for the 10 repetitions (Case a) and 5 repetitions (Case b).

### 3. 6 Functional and Pathway Analysis

Functional and pathway analysis has been performed on these 120 genes for further validation of our results. We have used DAVID [50] and STRING [51] for the functional annotation of the association between these genes. Functional annotation of these 120 genes clustered as 8 functionally related groups. Most of the genes in each group are related with prostate cancer and the most known genes in relation with prostate cancer with the clusters of genes can be found in S7 Table.

To find the prostate cancer related pathways, we performed a pathway analysis using databases like DAVID [50], KEGG [52], FatiGO [53]. The resulted pathways with significant p-value are given in Table 9. A Pubmed search confirmed that all the resulting pathways are related to prostate cancer. Our analysis also identified several other genes that have no related publications in relation with prostate cancer.

**Table 9:** The top 14 resulted pathways from pathway analysis.

Pathway name	Pathway Classification	P-value	Reference
Integrin signalling pathway	Cell communication	1.03E-08	[85, 95]
Smooth Muscle Contraction	Organismal Systems; Circulatory system	5.98E-08	[96, 97]
Oxytocin signalling pathway	Organismal Systems; Endocrine system	1.23E-08	[98–100]
Collagen biosynthesis and modifying enzymes	Metabolism; Amino acid metabolism	1.13E-07	[88, 101]
Axon guidance	Development	1.34E-06	[102]
Gap junction trafficking	Cell communication	3.12E-06	[103, 104]
Protein digestion and absorption	Organismal Systems; Digestive system	3.6E-06	[101]
Ras activation	Regulation of translation and transcription	3.46E-05	[105, 106]
regulation of pgc-1a	Cell motility	3.61E-05	[107]
Assembly of collagen fibrils and other multimeric structures	Metabolism	3.68E-05	[108]
CREB phosphorylation	Metabolism; Energy metabolism	6.31E-05	[109]
Syndecan-1-mediated signalling events	Genetic Information Processing	6.3E-05	[110]
NCAM1 interactions	Signal Transduction	6.72E-05	[111]
regulators of bone mineralization	Metabolism	6.7E-05	[112]

**Pathway Name** is the name of the pathways. **Pathway Classification** is the class of each pathway. **P-value** is the respective p-value for each pathway. **Reference** is the papers which show the relation of each pathway with prostate cancer.

## Discussion

The microarray technology has a tremendous impact on cancer research in assessing the presence of cancer cells in patient tissues. The rapid acquisition of microarray data makes it possible to integrate this large amount of data across a range of platforms. In this study, we identified robust cancer gene expression signatures common to all datasets. The comparison of our proposed method with individual study results highlights the advantages of meta-analysis over individual studies. The comparison of our method with one of the state of the art methods shows the robustness of this method.

Results of *(α*,*β)-k-*Feature Set selection for each individual dataset provide signatures of reasonable size capable of discriminating between primary tumours and normal samples. However even though individual signatures consist of a large number of features, the number of common genes is limited to seven and is too little for further analysis. The result of Coloured *(α*,*β)-k-*Feature Set problem shows a vast difference in the number of resulting genes and is reliable for further analysis. The combined dataset contains 10729 out of 16157 combinations of probes from three or more datasets. That confirms we get a good coverage on all the datasets by using the proposed method of integration. Furthermore, the Coloured *(α*,*β)-k-*Feature Set problem results show that around 2272 out of 3190 resulted features cover four or more datasets.

Even though the *(α*,*β)-k-*Feature Set methodology and *t*-test provided good results on individual datasets, a large number of genes have been eliminated from the common set of genes which may include potential biomarkers. So when we performed the data integration instead of considering common genes on individual dataset results, the number of resulting genes is significantly increased. This shows that the proposed method makes it possible to uncover robust biomarkers by increasing the sample size to a sufficient level and helps to capture the consistent features that might have been masked because of the limitations of individual studies. As we have a reasonable number of genes, they can also provide more information about prostate cancer.

The result of Coloured *(α*,*β)-k-*Feature Set problem evidences a high level of agreement with the top listed genes of RankProd result, where almost 80% of our signature is included in RankProd’s result. However, RankProd results are considerably larger in size, hindering interpretation. Additionally, as mentioned before, RankProd artificially reduces the rank of any gene with missing values (escalating its position to the significant side of the list), which: i) restricts applicability to the genes represented in all platforms, and ii) introduces non-linear rank scaling in the presence of scattered missing values. In contrast, Coloured *(α*,*β)-k-*Feature Set methodology automatically deals with any amount of missing values (that is, a gene may not be present in a dataset but still be significant to explain a large number of sample pairs in the other datasets), providing a more reliable result. Although not used in our investigation, Coloured *(α*,*β)-k-*Feature Set methodology allows for weights to be assigned to genes and samples independently, and account for an external perceived relative confidence in each experimental condition, if so desired.

The sensitivity analysis shows a high level of consistency with the original solution. Each step of the sensitivity analysis confirms that the proposed method is not relying on a single or a small set of genes. The results of the analysis also show that the significance of the gene is not dependent on a single dataset. The consistency of the results shows the robustness of our proposed method and validates the findings.

It is not surprising that most of the signature genes have been reported to be related with prostate cancer. For instance, AMACR [54–59], HPN [60–62], SOX4 [63–67], DAXX [68, 69], EPB41L3 [70–72], CXCR3 [73–79], TGFB3 [80, 81], EEF2 [82, 83] are the most well-known biomarkers for prostate cancer. As defined by the Gene Ontology Consortium, most of the resulted genes are involved in cell cycle (MYH11), regulation of transcription (SOX4, SMARCC2, ZIM2, PDLIM5, ZNF217, PSIPI, ACRC, PEG3, TAF1, ZMYM3), receptor activity (JAM3, TAPBP, COL4A5, CXCR3, COL4A6, HPN, COL9A2, PTPRN2, COL6A1) and other biological activities like transportation, cell adhesion and cell organisation (the list of genes with the related literature references can be found in S8 Table).

Most of the genes mentioned above and in S8 Table are highly correlated with prostate cancer. However we could find only some of them in the individual dataset results. We have also uncovered genes which participate in the same pathway class as genes related to prostate cancer, but have not yet been reported in relation to prostate cancer. For instance, the gene NUDT3 is not yet reported in relation to prostate cancer, but NUDT3 participates in the Collagen biosynthesis and modifying enzymes pathway which has been identified as a prostate cancer related pathway [84]. This indirectly suggests that this gene may also have some influence on cancer development.

Interestingly, the most significant pathway overrepresented in our results is the Integrin signalling pathway and focal adhesion. Integrins are transmembrane receptors and play an important role in cell survival, proliferation, migration, gene expression, and activation of growth factor receptors. Studies show that integrins are down regulated in the transition from normal prostate tissue to primary localized prostate cancer [85]. From our resulted list of genes COL4A5, COL4A6, COL6A1 and ITGB1BP2 are participating in integrin signalling pathway.

Smooth muscles found in the walls of reproductive tract of male and female which is made up of actin and myosin, together have the capacity to contract and relax. The prostate helps to control urine flow and ejaculations, via contractions and relaxation of its smooth muscle layers. The uncontrolled contraction of prostate smooth muscle may result in urinary tract problems in addition to prostate growth [86]. The smooth muscle contraction pathway has already been reported related with prostate cancer [87]. From our list of genes MYH11, MYL6, MYL6B and GUCY1A3 are related with smooth muscle contraction.

Collagen biosynthesis is the biosynthetic pathway responsible for collagen production. Studies have shown that the Gleason sum is increasing with decreasing cancer collagen content [88]. From our list of genes TGFB, COL4A5, COL4A6, COL6A1 and COL9A2 are related with collagen biosynthesis.

The outcomes of our work support the claim that the proposed method is a viable meta-analysis method for feature selection. The functional and pathway analysis results show that the Coloured (*α*,*β*)-*k*-Feature Set approach is capable of uncovering genes with significant and biologically relevant functions that other, non-integrative methods fail to identify.

## Conclusion

We have presented the Coloured (*α*,*β*)-*k*-Feature Set problem as a combinatorial optimisation approach for multi-platform integration analysis without the need for normalisation of the data between datasets. The results indicate that the method is capable of providing highly significant signatures, even where the individual datasets before integration are small and thus lacking informational content. The method is generic and does not depend on inherent properties of gene expression data, allowing it to be potentially applied to any dataset where the notions of features, class based classification and equality between feature values is meaningful. In applying this methodology to an integrated prostate cancer dataset we have identified potential novel prostate cancer associated pathways and genes. As the number of cancer datasets increases we will be able to use this novel and robust method to combine more cancer datasets and identify more candidate pathways and genes.

## Supporting Information

S1 Table List of combination of probes. List of combination of probes resulted by applying the integration method. The probes are selected according to the conditions and the selected probe ID is given with the corresponding dataset name. The table also contains the gene names correspond to each combination of probe. (XLS)
(XLS)

S2 Table Individual *(α*,*β)-k*-Feature Set problem Results. The list of genes resulted by applying *(α*,*β)-k*-Feature Set methodology on individual datasets. Single XLS contains six worksheets, one for each dataset result. The worksheets are names according to the dataset name.
(XLS)

S3 Table *t*-test result. List of genes resulted after applying *t*-test on each dataset. XLS contains six worksheets, for each dataset. Each worksheet is named according to the dataset name.
(XLS)

S4 Table Coloured *(α*,*β)-k*-Feature Set problem Result. The list of 3190 combined probes and 1788 genes resulted after applying the Coloured *(α*,*β)-k*-Feature Set methodology on the combined dataset. Also another worksheet with the annotation result of 1788 genes.
(XLS)

S5 Table List of common genes resulted from Coloured (α,β)-k-Feature Set problem. An XLS file contains the list of 120 genes which are common in all the six datasets and the annotation details with the heatmap in sheet 1 and the list of 186 genes common in five or more datasets with annotation details and heatmap in sheet 2.
(XLS)

S6 Table RankProd Result. The list of RankProd resulted genes with related rank, pfp and p-value.
(XLS)

S7 Table Result of functional analysis. The details of eight clusters resulted from functional analysis using DAVID.
(XLS)

S8 Table List of 120 genes with related literature references. Word document with the list of 120 genes which are common in all six datasets and the related literature references
(DOCX)
