# Supplementary material for: A New Combinatorial Optimization Approach for Integrated Feature Selection Using Different Datasets: A Prostate Cancer Transcriptomic Study
Source: PLoS One. 2015 Jun 24;10(6):e0127702. doi: 10.1371/journal.pone.0127702 (PMC4480358; doi:10.1371/journal.pone.0127702)
Supplement: S8 Table — Word document with the list of 120 genes which are common in all six datasets and the related literature references (DOCX) [file pone.0127702.s008.docx]

| Gene Name | Reference | Details |
| --- | --- | --- |
| AMACR | [[1-11](#_ENREF_1)] [[1](#_ENREF_1), [3](#_ENREF_3), [5](#_ENREF_5), [12-40](#_ENREF_12)] | Plays an important role in catalytic activity. Increased levels of AMACR protein and activity are associated with prostate cancer. Around 10 different variants of human AMACR which arise from alternative mRNA splicing have been identified from prostate cancer tissues. |
| ERG | [[1](#_ENREF_1), [13](#_ENREF_13), [14](#_ENREF_14), [35](#_ENREF_35), [41-124](#_ENREF_41)] |  |
| HPN | [[125-147](#_ENREF_125)] | Plays an important role in cell growth and maintenance of cell morphology. A major 11 locus haplotype of HPN is associated with prostate cancer. |
| SOX4 | [[148-155](#_ENREF_148)] [[90](#_ENREF_90), [156-162](#_ENREF_156)] | An important transcriptional activator. Studies have been shown that SOX4 is overexpressed in prostate cancer samples. |
| APOC1 | [[163-165](#_ENREF_163)] |  |
| DAXX | [[166-172](#_ENREF_166)] | Plays a role as transcription repressor. DAXX is frequently up regulated in prostate cancer samples; the expression level is positively correlated with the Gleason score, also leading to a delay in mitotic progression. |
| EPB41L3 | [[173-175](#_ENREF_173)] | Plays a role in protein binding. Studies have shown that it is frequently down regulated in prostate cancer. |
| CXCR3 | [[176-182](#_ENREF_176)] | Plays an important role as a mediator for proliferation, survival and angiogenic activity of human mesangial cells (HMC) through a heterotrimeric G-protein signalling pathway. Studies have shown that CXCR3 can promote prostate cancer through cell migration. |
| TGFB3 | [[183-186](#_ENREF_183)] | Involved in embryogenesis and cell differentiation. Studies have shown that TGFB3 is over expressed in prostate cancer. |
| TPM2 | [[187](#_ENREF_187)] [[188](#_ENREF_188)] | Smooth muscle contraction is regulated by interaction with caldesmon. It has been shown that this gene is under expressed in prostate cancer. |
| EEF2 | [[189-191](#_ENREF_189)] | Catalyses the GTP-dependent ribosomal translocation step during translation elongation. It has been shown that this gene is over expressed in prostate cancer. |
| ACAD8 | [[192](#_ENREF_192)] | Plays a role in transcriptional coactivation within the ARC complex. ACAD8 mutation has been identified in the case of prostate cancer. |
| COL4A5 | [[193](#_ENREF_193)] | Type IV collagen is the major structural component of glomerular basement membranes. An altered expression of COL4A5 has been found in prostate cancer. |
| COL4A6 | [[187](#_ENREF_187)] | Type IV collagen is the major structural component of glomerular basement membranes. It has been reported that this gene is down regulated in prostate cancer. |
| COL6A1 | [[193](#_ENREF_193)] | Collagen VI acts as a cell-binding protein. It has been reported that this gene is down regulated in prostate cancer. |
| CRYAB | [[194](#_ENREF_194)] | CRYAB help to prevent the aggregation of various proteins under a wide range of stress conditions. It is reported that CRYAB is over expressed in prostate cancer. |
| GCNT1 | [[195](#_ENREF_195), [196](#_ENREF_196)] | Glycosyltransferase that catalyzes the transfer of an N-acetylglucosamine moiety onto mucin-type core 1 O-glycan to form the branched mucin-type core 2 O-glycan. It is shown that GCNT1 is over expressed in prostate cancer. |
| GUCY1A3 | [[197](#_ENREF_197)] |  |
| PTPRN2 | [[183](#_ENREF_183)] |  |
| SVIL | [[152](#_ENREF_152)] |  |
| UGDH | [[198-200](#_ENREF_198)] |  |
| VPS26B |  | A complex required to retrieve lysosomal enzyme receptors from endosomes to the trans-Golgi network. Could not find any references related with prostate cancer. But VPS26B has already been reported related with breast, bladder, colon, rectal and gastric cancers. |
| VPS52 |  | VPS52 is involved in retrograde transport of early and late endosomes to the late Golgi. Could not find any relevant information related with prostate cancer. But it has been reported related with other types of cancers like head, neck, ovarian, breast, colorectal and lung cancer. |
| WBP1 |  | WBP1 is putatively involved in protein-protein interaction. It has been reported related with cancers like breast, ovarian, colon, rectal, etc. |
| WBP4 |  | Promotes pre-mRNA splicing. WBP4 has been reported related with other type of cancers rather than prostate cancer. |
| WDR46 |  |  |
| ZBTB22 |  |  |
| ZIM2 |  |  |
| ZMYM3 |  |  |
| ZNF217 | [[201](#_ENREF_201), [202](#_ENREF_202)] |  |
| ACRC |  |  |
| ACSM1 |  |  |
| ANK3 | [[203](#_ENREF_203)] |  |
| ATG4A |  |  |
| B3GALT4 |  |  |
| BCYRN1 |  |  |
| C11orf52 |  |  |
| C1QTNF3 |  |  |
| C1QTNF3-AMACR |  |  |
| C9orf91 |  |  |
| CA9 | [[204](#_ENREF_204)] |  |
| CALM1 |  |  |
| CAMK2G |  |  |
| CAMKK2 | [[205-208](#_ENREF_205)] |  |
| CELF2 |  |  |
| CNPY2 |  |  |
| COL9A2 |  |  |
| CRYZL1 |  |  |
| CXorf49 |  |  |
| CXorf49B |  |  |
| DDT | [[209](#_ENREF_209)] |  |
| DDTL |  |  |
| DONSON |  |  |
| DPT |  |  |
| ENC1 |  |  |
| FLJ46446 |  |  |
| GALNT3 |  |  |
| GART |  |  |
| GBP2 |  |  |
| GJB1 |  |  |
| GLT25D2 |  |  |
| GSTT2 | [[209](#_ENREF_209)] |  |
| HMGN4 |  |  |
| HSPB2 |  |  |
| HSPB2-C11orf52 |  |  |
| ICA1 |  |  |
| INGX |  |  |
| INO80B |  |  |
| INO80B-WBP1 |  |  |
| ITGB1BP2 |  |  |
| ITSN1 |  |  |
| JAM3 |  |  |
| KIAA0430 |  |  |
| KIAA0913 |  |  |
| LDHB | [[210](#_ENREF_210)] |  |
| LPIN1 |  |  |
| MAP7 |  | MAP7 plays an important role during reorganization of microtubules during polarization and differentiation of epithelial cells. Could not find any references related with prostate cancer but it has been reported related with other types of cancers. |
| ME1 | [[211](#_ENREF_211)] | ME1 functions for reversible oxidative decarboxylation of malate, links the glycolytic and citric acid cycles. It has been reported that this gene is related with prostate cancer. |
| MIMT1 |  | It is an imprinted gene, which is paternally expressed |
| MOGS |  |  |
| MYH11 |  |  |
| MYL6 |  | Regulatory light chain of myosin. Participate in smooth muscle contraction and relaxation. |
| MYL6B |  | Regulatory light chain of myosin. Participate in smooth muscle contraction and relaxation. |
| MYO6 | [[116](#_ENREF_116), [212](#_ENREF_212), [213](#_ENREF_213)] | Plays an important role in vesicular membrane trafficking and cell migration. It has been shown that the Golgi apparatus in prostate cancer cells differs from the normal Golgi by elevated levels of MYO6. |
| NCAPD3 |  | NCAPD3 is involved in physical rigidity of the chromatid axis. |
| NDE1 | [[214](#_ENREF_214)] | NDE1 helps to regulate the production of neurons by controlling the orientation of the mitotic spindle during division of cortical neuronal progenitors of the proliferative ventricular zone of the brain. It has been shown that NDE1 along with TWIST1 promotes metastasis. |
| NDST2 | [[215](#_ENREF_215)] | Essential bi functional enzyme that catalyses both the N-deacetylation and the N-sulfation of glucosamine of the glycosaminoglycan in heparan sulfate. It is reported that NDST2 which is a part of HS metabolic system is involved in prostate carcinogenesis. |
| NONO |  | DNA- and RNA binding protein, involved in several nuclear processes. |
| NUDT3 |  | Plays a role in signal transduction. |
| OGT |  |  |
| OPTN |  |  |
| PAN2 |  |  |
| PCSK6 |  |  |
| PDLIM5 | [[216](#_ENREF_216)] |  |
| PDPN |  |  |
| PEG3 |  |  |
| PEG3-AS1 |  |  |
| PFDN6 |  |  |
| PLP2 |  |  |
| PPAP2B |  |  |
| PPM1H |  |  |
| PPP1R3C |  |  |
| PRICKLE3 |  |  |
| PSIP1 |  |  |
| PSMD10 |  |  |
| RBPMS |  |  |
| RCAN2 |  |  |
| RGL2 |  |  |
| RPS10 |  |  |
| RPS10-NUDT3 |  |  |
| RPS18 |  |  |
| SCN1B |  |  |
| SLC25A6 |  |  |
| SLC7A1 |  |  |
| SMARCC2 |  |  |
| SNORD37 |  |  |
| SON |  |  |
| SPG20 |  |  |
| TAF1 | [[217](#_ENREF_217)] |  |
| TARP | [[218-224](#_ENREF_218)] |  |
| TAPBP |  |  |
| THYN1 |  |  |

1. Andrews C, Humphrey PA. Utility of ERG versus AMACR expression in diagnosis of minimal adenocarcinoma of the prostate in needle biopsy tissue. The American journal of surgical pathology. 2014;38(7):1007-12. Epub 2014/04/08. doi: 10.1097/PAS.0000000000000205. PubMed PMID: 24705308.

2. Bansal N, Davis S, Tereshchenko I, Budak-Alpdogan T, Zhong H, Stein MN, et al. Enrichment of human prostate cancer cells with tumor initiating properties in mouse and zebrafish xenografts by differential adhesion. Prostate. 2014;74(2):187-200. doi: 10.1002/pros.22740. PubMed PMID: 24154958; PubMed Central PMCID: PMC3939797.

3. Carnell AJ, Kirk R, Smith M, McKenna S, Lian L-Y, Gibson R. Inhibition of Human alpha-Methylacyl CoA Racemase (AMACR): a Target for Prostate Cancer. ChemMedChem. 2013. Epub 2013/08/10. doi: 10.1002/cmdc.201300179. PubMed PMID: 23929631.

4. Erdmann K, Kaulke K, Thomae C, Huebner D, Sergon M, Froehner M, et al. Elevated expression of prostate cancer-associated genes is linked to down-regulation of microRNAs. BMC cancer. 2014;14:82. doi: 10.1186/1471-2407-14-82. PubMed PMID: 24517338; PubMed Central PMCID: PMC3923006.

5. Jiang N, Zhu S, Chen J, Niu Y, Zhou L. A-methylacyl-CoA racemase (AMACR) and prostate-cancer risk: a meta-analysis of 4,385 participants. PLoS One. 2013;8(10):e74386. Epub 2013/10/17. doi: 10.1371/journal.pone.0074386. PubMed PMID: 24130666; PubMed Central PMCID: PMC3794046.

6. Kiflemariam S, Mignardi M, Ali MA, Bergh A, Nilsson M, Sjöblom T. In situ sequencing identifies TMPRSS2–ERG fusion transcripts, somatic point mutations and gene expression levels in prostate cancers. The Journal of Pathology. 2014;234(2):253-61. doi: 10.1002/path.4392.

7. Lee S-J, Joung JY, Yoon H, Kim JE, Park WS, Seo HK, et al. Genetic Variations of &#x3b1;-Methylacyl-CoA Racemase Are Associated with Sporadic Prostate Cancer Risk in Ethnically Homogenous Koreans. BioMed Research International. 2013;2013:11. doi: 10.1155/2013/394285.

8. Lippolis G, Edsjo A, Helczynski L, Bjartell A, Overgaard N. Automatic registration of multi-modal microscopy images for integrative analysis of prostate tissue sections. BMC Cancer. 2013;13(1):408. PubMed PMID: doi:10.1186/1471-2407-13-408.

9. Mahdian R, Nodouzi V, Asgari M, Rezaie M, Alizadeh J, Yousefi B, et al. Expression profile of MAGI2 gene as a novel biomarker in combination with major deregulated genes in prostate cancer. Mol Biol Rep. 2014;41(9):6125-31. doi: 10.1007/s11033-014-3491-0.

10. Paziewska A, Dabrowska M, Goryca K, Antoniewicz A, Dobruch J, Mikula M, et al. DNA methylation status is more reliable than gene expression at detecting cancer in prostate biopsy. Br J Cancer. 2014;111(4):781-9. doi: 10.1038/bjc.2014.337.

11. Yu YP, Ding Y, Chen Z, Liu S, Michalopoulos A, Chen R, et al. Novel Fusion Transcripts Associate with Progressive Prostate Cancer. The American Journal of Pathology. 2014;184(10):2840-9. doi: <http://dx.doi.org/10.1016/j.ajpath.2014.06.025>.

12. Ananthanarayanan V, Deaton RJ, Yang XJ, Pins MR, Gann PH. Alpha-methylacyl-CoA racemase (AMACR) expression in normal prostatic glands and high-grade prostatic intraepithelial neoplasia (HGPIN): Association with diagnosis of prostate cancer. The Prostate. 2005;63(4):341-6. doi: 10.1002/pros.20196. PubMed PMID: 15602744.

13. Svensson MA, Perner S, Ohlson AL, Day JR, Groskopf J, Kirsten R, et al. A comparative study of ERG status assessment on DNA, mRNA, and protein levels using unique samples from a Swedish biopsy cohort. Applied immunohistochemistry & molecular morphology : AIMM / official publication of the Society for Applied Immunohistochemistry. 2014;22(2):136-41. Epub 2014/02/13. doi: 10.1097/PDM.0b013e31829e0484. PubMed PMID: 24517914.

14. Qi M, Yang X, Zhang F, Lin T, Sun X, Li Y, et al. ERG rearrangement is associated with prostate cancer-related death in Chinese prostate cancer patients. PloS one. 2014;9(2):e84959. Epub 2014/02/12. doi: 10.1371/journal.pone.0084959. PubMed PMID: 24516518; PubMed Central PMCID: PMC3917829.

15. Iczkowski KA. Paneth cell-like change in benign prostate can account for P504S (AMACR) reactivity. International journal of clinical and experimental pathology. 2014;7(6):3454-5. Epub 2014/07/18. PubMed PMID: 25031776; PubMed Central PMCID: PMC4097236.

16. Ozgur T, Atik E, Hakverdi S, Yaldiz M. The expressions of AMACR and iNOS in prostate adenocarcinomas. Pakistan journal of medical sciences. 2013;29(2):610-3. Epub 2013/12/20. PubMed PMID: 24353588; PubMed Central PMCID: PMC3809268.

17. Yamada H, Tsuzuki T, Maeda N, Yamauchi Y, Yoshida S, Ishida R, et al. Alpha methylacyl-CoA racemase (AMACR) in prostate adenocarcinomas from Japanese patients: is AMACR a "race"-dependent marker? The Prostate. 2013;73(1):54-9. Epub 2012/05/18. doi: 10.1002/pros.22539. PubMed PMID: 22593005.

18. Vanguri VK, Woda BA, Jiang Z. Sensitivity of P504S/alpha-methylacyl-CoA racemase (AMACR) immunohistochemistry for the detection of prostate carcinoma on stored needle biopsies. Applied immunohistochemistry & molecular morphology : AIMM / official publication of the Society for Applied Immunohistochemistry. 2006;14(3):365-8. Epub 2006/08/26. PubMed PMID: 16932031.

19. Ananthanarayanan V, Deaton RJ, Yang XJ, Pins MR, Gann PH. Alpha-methylacyl-CoA racemase (AMACR) expression in normal prostatic glands and high-grade prostatic intraepithelial neoplasia (HGPIN): association with diagnosis of prostate cancer. Prostate. 2005;63(4):341-6. Epub 2004/12/17. doi: 10.1002/pros.20196. PubMed PMID: 15602744.

20. Hameed O, Humphrey PA. p63/AMACR antibody cocktail restaining of prostate needle biopsy tissues after transfer to charged slides: a viable approach in the diagnosis of small atypical foci that are lost on block sectioning. American journal of clinical pathology. 2005;124(5):708-15. Epub 2005/10/06. doi: 10.1309/JXK1-BVAT-GBVN-Q9J9. PubMed PMID: 16203278.

21. Jiang Z, Li C, Fischer A, Dresser K, Woda BA. Using an AMACR (P504S)/34betaE12/p63 cocktail for the detection of small focal prostate carcinoma in needle biopsy specimens. American journal of clinical pathology. 2005;123(2):231-6. Epub 2005/04/22. PubMed PMID: 15842047.

22. Browne T-J, Hirsch MS, Brodsky G, Welch WR, Loda MF, Rubin MA. Prospective evaluation of AMACR (P504S) and basal cell markers in the assessment of routine prostate needle biopsy specimens. Hum Pathol. 2004;35(12):1462-8. Epub 2004/12/25. PubMed PMID: 15619204.

23. Gologan A, Bastacky S, McHale T, Yu J, Cai C, Monzon-Bordonaba F, et al. Age-associated changes in alpha-methyl CoA racemase (AMACR) expression in nonneoplastic prostatic tissues. The American journal of surgical pathology. 2005;29(11):1435-41. Epub 2005/10/15. PubMed PMID: 16224209.

24. Thornburg T, Turner AR, Chen YQ, Vitolins M, Chang B, Xu J. Phytanic acid, AMACR and prostate cancer risk. Future oncology. 2006;2(2):213-23. Epub 2006/03/28. doi: 10.2217/14796694.2.2.213. PubMed PMID: 16563090.

25. Walter B, Weiss T, Hofstadter F, Gaumann A, Hartmann A, Rogenhofer S, et al. Utility of immunohistochemistry markers in the interpretation of post-high-intensive focussed ultrasound prostate biopsy cores. World journal of urology. 2013;31(5):1129-33. Epub 2012/02/22. doi: 10.1007/s00345-012-0838-9. PubMed PMID: 22350516.

26. Lloyd MD, Yevglevskis M, Lee GL, Wood PJ, Threadgill MD, Woodman TJ. alpha-Methylacyl-CoA racemase (AMACR): metabolic enzyme, drug metabolizer and cancer marker P504S. Progress in lipid research. 2013;52(2):220-30. Epub 2013/02/05. doi: 10.1016/j.plipres.2013.01.001. PubMed PMID: 23376124.

27. Lee S-J, Joung JY, Yoon H, Kim JE, Park WS, Seo HK, et al. Genetic variations of alpha -methylacyl-CoA racemase are associated with sporadic prostate cancer risk in ethnically homogenous Koreans. BioMed research international. 2013;2013:394285. Epub 2014/01/03. doi: 10.1155/2013/394285. PubMed PMID: 24383053; PubMed Central PMCID: PMC3870614.

28. Deng F-M, Zhao Y, Kong X, Lee P, Melamed J. Construction of tissue microarrays using pre-existing slides as source of tissue when paraffin blocks are unavailable. Journal of clinical pathology. 2013;66(7):627-9. Epub 2013/03/12. doi: 10.1136/jclinpath-2012-201171. PubMed PMID: 23476078.

29. Dabir PD, Ottosen P, Hoyer S, Hamilton-Dutoit S. Comparative analysis of three- and two-antibody cocktails to AMACR and basal cell markers for the immunohistochemical diagnosis of prostate carcinoma. Diagnostic pathology. 2012;7:81. Epub 2012/07/18. doi: 10.1186/1746-1596-7-81. PubMed PMID: 22800084; PubMed Central PMCID: PMC3434074.

30. Clouston D, Bolton D. In situ and intraductal epithelial proliferations of prostate: definitions and treatment implications. Part 1: Prostatic intraepithelial neoplasia. BJU international. 2012;109 Suppl 3:22-6. Epub 2012/04/04. doi: 10.1111/j.1464-410X.2012.11040.x. PubMed PMID: 22458488.

31. Barry M, Dhillon PK, Stampfer MJ, Perner S, Ma J, Giovannucci E, et al. alpha-Methylacyl-CoA racemase expression and lethal prostate cancer in the Physicians' Health Study and Health Professionals Follow-up Study. The Prostate. 2012;72(3):301-6. Epub 2011/06/30. doi: 10.1002/pros.21432. PubMed PMID: 21713964; PubMed Central PMCID: PMC3267640.

32. Al-Maghrebi M, Kehinde EO, Anim JT, Sheikh M. The role of combined measurement of tissue mRNA levels of AMACR and survivin in the diagnosis and risk stratification of patients with suspected prostate cancer. International urology and nephrology. 2012;44(6):1681-9. Epub 2012/06/22. doi: 10.1007/s11255-012-0220-2. PubMed PMID: 22718029.

33. Ouyang B, Leung Y-K, Wang V, Chung E, Levin L, Bracken B, et al. alpha-Methylacyl-CoA racemase spliced variants and their expression in normal and malignant prostate tissues. Urology. 2011;77(1):249 e1-7. Epub 2011/01/05. doi: 10.1016/j.urology.2010.08.005. PubMed PMID: 21195844; PubMed Central PMCID: PMC3051191.

34. Noske A, Zimmermann A-K, Caduff R, Varga Z, Fink D, Moch H, et al. Alpha-methylacyl-CoA racemase (AMACR) expression in epithelial ovarian cancer. Virchows Archiv : an international journal of pathology. 2011;459(1):91-7. Epub 2011/06/07. doi: 10.1007/s00428-011-1095-9. PubMed PMID: 21643692.

35. Sorensen KD, Orntoft TF. Discovery of prostate cancer biomarkers by microarray gene expression profiling. Expert review of molecular diagnostics. 2010;10(1):49-64. Epub 2009/12/18. doi: 10.1586/erm.09.74. PubMed PMID: 20014922.

36. Prior C, Guillen-Grima F, Robles JE, Rosell D, Fernandez-Montero JM, Agirre X, et al. Use of a combination of biomarkers in serum and urine to improve detection of prostate cancer. World journal of urology. 2010;28(6):681-6. Epub 2010/07/16. doi: 10.1007/s00345-010-0583-x. PubMed PMID: 20632177.

37. Ouazia D, Bearne SL. A continuous assay for alpha-methylacyl-coenzyme A racemase using circular dichroism. Analytical biochemistry. 2010;398(1):45-51. Epub 2009/10/27. doi: 10.1016/j.ab.2009.10.039. PubMed PMID: 19854148.

38. Jamaspishvili T, Kral M, Khomeriki I, Student V, Kolar Z, Bouchal J. Urine markers in monitoring for prostate cancer. Prostate cancer and prostatic diseases. 2010;13(1):12-9. Epub 2009/08/05. doi: 10.1038/pcan.2009.31. PubMed PMID: 19652665.

39. Takahara K, Azuma H, Sakamoto T, Kiyama S, Inamoto T, Ibuki N, et al. Conversion of prostate cancer from hormone independency to dependency due to AMACR inhibition: involvement of increased AR expression and decreased IGF1 expression. Anticancer research. 2009;29(7):2497-505. Epub 2009/07/15. PubMed PMID: 19596919.

40. Berretta R, Moscato P. Cancer biomarker discovery: the entropic hallmark. PloS one. 2010;5(8):e12262. Epub 2010/09/02. doi: 10.1371/journal.pone.0012262. PubMed PMID: 20805891; PubMed Central PMCID: PMC2923618.

41. Obinata D, Ito A, Fujiwara K, Takayama K-i, Ashikari D, Murata Y, et al. Pyrrole-imidazole polyamide targeted to break fusion sites in TMPRSS2 and ERG gene fusion represses prostate tumor growth. Cancer science. 2014;105(10):n/a-n/a. doi: 10.1111/cas.12493. PubMed PMID: 25088707.

42. Udager AM, Shi Y, Tomlins SA, Alva A, Siddiqui J, Cao X, et al. Frequent discordance between ERG gene rearrangement and ERG protein expression in a rapid autopsy cohort of patients with lethal, metastatic, castration-resistant prostate cancer. The Prostate. 2014;74(12):1199-208. doi: 10.1002/pros.22836.

43. Steurer S, Mayer PS, Adam M, Krohn A, Koop C, Ospina-Klinck D, et al. TMPRSS2-ERG Fusions Are Strongly Linked to Young Patient Age in Low-grade Prostate Cancer. European Urology. 2014;(0). doi: 10.1016/j.eururo.2014.06.027.

44. Rastogi A, Tan SH, Banerjee S, Sharad S, Kagan J, Srivastava S, et al. ERG Monoclonal Antibody in the Diagnosis and Biological Stratification of Prostate Cancer: Delineation of Minimal Epitope, Critical Residues for Binding, and Molecular Basis of Specificity. Monoclonal antibodies in immunodiagnosis and immunotherapy. 2014;33(4):201-8. Epub 2014/08/30. doi: 10.1089/mab.2014.0026. PubMed PMID: 25170998.

45. Rye MB, Bertilsson H, Drablos F, Angelsen A, Bathen TF, Tessem MB. Gene signatures ESC, MYC and ERG-fusion are early markers of a potentially dangerous subtype of prostate cancer. BMC medical genomics. 2014;7(1):50. Epub 2014/08/15. doi: 10.1186/1755-8794-7-50. PubMed PMID: 25115192.

46. Tallon L, Luangphakdy D, Ruffion A, Colombel M, Devonec M, Champetier D, et al. Comparative Evaluation of Urinary PCA3 and TMPRSS2: ERG Scores and Serum PHI in Predicting Prostate Cancer Aggressiveness. International journal of molecular sciences. 2014;15(8):13299-316. Epub 2014/08/01. doi: 10.3390/ijms150813299. PubMed PMID: 25079439.

47. Steurer S, Mayer PS, Adam M, Krohn A, Koop C, Ospina-Klinck D, et al. TMPRSS2-ERG Fusions Are Strongly Linked to Young Patient Age in Low-grade Prostate Cancer. Eur Urol. 2014. Epub 2014/07/13. doi: 10.1016/j.eururo.2014.06.027. PubMed PMID: 25015038.

48. Eguchi FC, Faria EF, Scapulatempo Neto C, Longatto-Filho A, Zanardo-Oliveira C, Taboga SR, et al. The role of TMPRSS2:ERG in molecular stratification of PCa and its association with tumor aggressiveness: a study in Brazilian patients. Scientific reports. 2014;4:5640. Epub 2014/07/11. doi: 10.1038/srep05640. PubMed PMID: 25007891; PubMed Central PMCID: PMC4090618.

49. Srivastava A, Price DK, Figg WD. Prostate tumor development and androgen receptor function alterations in a new mouse model with ERG overexpression and PTEN inactivation. Cancer biology & therapy. 2014;15(10):1293-5. Epub 2014/07/10. doi: 10.4161/cbt.29694. PubMed PMID: 25007053; PubMed Central PMCID: PMC4130720.

50. Delongchamps NB, Younes P, Denjean L, Zerbib M, Bories P-N. TMPRSS2-ERG fusion transcripts expression in patients referred for prostate biopsy: combining detection in urine and needle rinse material. World journal of urology. 2014. Epub 2014/07/06. doi: 10.1007/s00345-014-1359-5. PubMed PMID: 24997128.

51. Huang K-C, Alshalalfa M, Hegazy SA, Dolph M, Donnelly B, Bismar TA. The prognostic significance of combined ERG and androgen receptor expression in patients with prostate cancer managed by androgen deprivation therapy. Cancer biology & therapy. 2014;15(9):1120-8. Epub 2014/06/28. doi: 10.4161/cbt.29689. PubMed PMID: 24972028; PubMed Central PMCID: PMC4128855.

52. Velaeti S, Dimitriadis E, Kontogianni-Katsarou K, Savvani A, Sdrolia E, Pantazi G, et al. Detection of TMPRSS2-ERG fusion gene in benign prostatic hyperplasia. Tumour biology : the journal of the International Society for Oncodevelopmental Biology and Medicine. 2014. Epub 2014/06/26. doi: 10.1007/s13277-014-2250-0. PubMed PMID: 24961351.

53. Smith SC, Tomlins SA. Prostate Cancer SubtyPINg BiomarKers and Outcome: Is Clarity EmERGing? Clinical cancer research : an official journal of the American Association for Cancer Research. 2014. Epub 2014/06/20. doi: 10.1158/1078-0432.CCR-14-0818. PubMed PMID: 24944315.

54. Kiflemariam S, Mignardi M, Ali MA, Bergh A, Nilsson M, Sjoblom T. In situ sequencing identifies TMPRSS2-ERG fusion transcripts, somatic point mutations and gene expression levels in prostate cancers. The Journal of pathology. 2014;234(2):253-61. Epub 2014/06/17. doi: 10.1002/path.4392. PubMed PMID: 24931216.

55. Huang KC, Dolph M, Donnelly B, Bismar TA. ERG expression is associated with increased risk of biochemical relapse following radical prostatectomy in early onset prostate cancer. Clinical & translational oncology : official publication of the Federation of Spanish Oncology Societies and of the National Cancer Institute of Mexico. 2014;16(11):973-9. Epub 2014/05/07. doi: 10.1007/s12094-014-1182-x. PubMed PMID: 24796295.

56. Krohn A, Freudenthaler F, Harasimowicz S, Kluth M, Fuchs S, Burkhardt L, et al. Heterogeneity and chronology of PTEN deletion and ERG fusion in prostate cancer. Modern pathology : an official journal of the United States and Canadian Academy of Pathology, Inc. 2014;27(12):1612-20. Epub 2014/04/26. doi: 10.1038/modpathol.2014.70. PubMed PMID: 24762546.

57. Hagglof C, Hammarsten P, Stromvall K, Egevad L, Josefsson A, Stattin P, et al. TMPRSS2-ERG expression predicts prostate cancer survival and associates with stromal biomarkers. PloS one. 2014;9(2):e86824. Epub 2014/02/08. doi: 10.1371/journal.pone.0086824. PubMed PMID: 24505269; PubMed Central PMCID: PMC3914792.

58. Blum R, Gupta R, Burger PE, Ontiveros CS, Salm SN, Xiong X, et al. Molecular signatures of prostate stem cells reveal novel signaling pathways and provide insights into prostate cancer. PloS one. 2009;4(5):e5722. Epub 2009/05/30. doi: 10.1371/journal.pone.0005722. PubMed PMID: 19478945; PubMed Central PMCID: PMC2684642.

59. Camoes MJ, Paulo P, Ribeiro FR, Barros-Silva JD, Almeida M, Costa VL, et al. Potential downstream target genes of aberrant ETS transcription factors are differentially affected in Ewing's sarcoma and prostate carcinoma. PloS one. 2012;7(11):e49819. Epub 2012/11/28. doi: 10.1371/journal.pone.0049819. PubMed PMID: 23185447; PubMed Central PMCID: PMC3501462.

60. Casey OM, Fang L, Hynes PG, Abou-Kheir WG, Martin PL, Tillman HS, et al. TMPRSS2- driven ERG expression in vivo increases self-renewal and maintains expression in a castration resistant subpopulation. PloS one. 2012;7(7):e41668. Epub 2012/08/04. doi: 10.1371/journal.pone.0041668. PubMed PMID: 22860005; PubMed Central PMCID: PMC3408501.

61. Chatterjee P, Choudhary GS, Sharma A, Singh K, Heston WD, Ciezki J, et al. PARP inhibition sensitizes to low dose-rate radiation TMPRSS2-ERG fusion gene-expressing and PTEN-deficient prostate cancer cells. PloS one. 2013;8(4):e60408. Epub 2013/04/09. doi: 10.1371/journal.pone.0060408. PubMed PMID: 23565244; PubMed Central PMCID: PMC3614551.

62. Chiu Y-T, Liu J, Tang K, Wong Y-C, Khanna KK, Ling M-T. Inactivation of ATM/ATR DNA damage checkpoint promotes androgen induced chromosomal instability in prostate epithelial cells. PloS one. 2012;7(12):e51108. Epub 2012/12/29. doi: 10.1371/journal.pone.0051108. PubMed PMID: 23272087; PubMed Central PMCID: PMC3525593.

63. Finones RR, Yeargin J, Lee M, Kaur AP, Cheng C, Sun P, et al. Early human prostate adenocarcinomas harbor androgen-independent cancer cells. PloS one. 2013;8(9):e74438. Epub 2013/10/03. doi: 10.1371/journal.pone.0074438. PubMed PMID: 24086346; PubMed Central PMCID: PMC3783414.

64. Kacprzyk LA, Laible M, Andrasiuk T, Brase JC, Borno ST, Falth M, et al. ERG induces epigenetic activation of Tudor domain-containing protein 1 (TDRD1) in ERG rearrangement-positive prostate cancer. PloS one. 2013;8(3):e59976. Epub 2013/04/05. doi: 10.1371/journal.pone.0059976. PubMed PMID: 23555854; PubMed Central PMCID: PMC3612037.

65. Ketola K, Kallioniemi O, Iljin K. Chemical biology drug sensitivity screen identifies sunitinib as synergistic agent with disulfiram in prostate cancer cells. PloS one. 2012;7(12):e51470. Epub 2012/12/20. doi: 10.1371/journal.pone.0051470. PubMed PMID: 23251544; PubMed Central PMCID: PMC3520796.

66. Kirby BJ, Jodari M, Loftus MS, Gakhar G, Pratt ED, Chanel-Vos C, et al. Functional characterization of circulating tumor cells with a prostate-cancer-specific microfluidic device. PloS one. 2012;7(4):e35976. Epub 2012/05/05. doi: 10.1371/journal.pone.0035976. PubMed PMID: 22558290; PubMed Central PMCID: PMC3338784.

67. Kunderfranco P, Mello-Grand M, Cangemi R, Pellini S, Mensah A, Albertini V, et al. ETS transcription factors control transcription of EZH2 and epigenetic silencing of the tumor suppressor gene Nkx3.1 in prostate cancer. PloS one. 2010;5(5):e10547. Epub 2010/05/19. doi: 10.1371/journal.pone.0010547. PubMed PMID: 20479932; PubMed Central PMCID: PMC2866657.

68. Leshem O, Madar S, Kogan-Sakin I, Kamer I, Goldstein I, Brosh R, et al. TMPRSS2/ERG promotes epithelial to mesenchymal transition through the ZEB1/ZEB2 axis in a prostate cancer model. PloS one. 2011;6(7):e21650. Epub 2011/07/13. doi: 10.1371/journal.pone.0021650. PubMed PMID: 21747944; PubMed Central PMCID: PMC3128608.

69. Magistroni V, Mologni L, Sanselicio S, Reid JF, Redaelli S, Piazza R, et al. ERG deregulation induces PIM1 over-expression and aneuploidy in prostate epithelial cells. PloS one. 2011;6(11):e28162. Epub 2011/12/06. doi: 10.1371/journal.pone.0028162. PubMed PMID: 22140532; PubMed Central PMCID: PMC3227636.

70. Massoner P, Kugler KG, Unterberger K, Kuner R, Mueller LAJ, Falth M, et al. Characterization of transcriptional changes in ERG rearrangement-positive prostate cancer identifies the regulation of metabolic sensors such as neuropeptide Y. PloS one. 2013;8(2):e55207. Epub 2013/02/08. doi: 10.1371/journal.pone.0055207. PubMed PMID: 23390522; PubMed Central PMCID: PMC3563644.

71. Qu X, Randhawa G, Friedman C, Kurland BF, Glaskova L, Coleman I, et al. A three-marker FISH panel detects more genetic aberrations of AR, PTEN and TMPRSS2/ERG in castration-resistant or metastatic prostate cancers than in primary prostate tumors. PloS one. 2013;8(9):e74671. Epub 2013/10/08. doi: 10.1371/journal.pone.0074671. PubMed PMID: 24098661; PubMed Central PMCID: PMC3787014.

72. Rahim S, Beauchamp EM, Kong Y, Brown ML, Toretsky JA, Uren A. YK-4-279 inhibits ERG and ETV1 mediated prostate cancer cell invasion. PloS one. 2011;6(4):e19343. Epub 2011/05/12. doi: 10.1371/journal.pone.0019343. PubMed PMID: 21559405; PubMed Central PMCID: PMC3084826.

73. Ribeiro FR, Paulo P, Costa VL, Barros-Silva JD, Ramalho-Carvalho J, Jeronimo C, et al. Cysteine-rich secretory protein-3 (CRISP3) is strongly up-regulated in prostate carcinomas with the TMPRSS2-ERG fusion gene. PloS one. 2011;6(7):e22317. Epub 2011/08/05. doi: 10.1371/journal.pone.0022317. PubMed PMID: 21814574; PubMed Central PMCID: PMC3141037.

74. Shao L, Zhou Z, Cai Y, Castro P, Dakhov O, Shi P, et al. Celastrol suppresses tumor cell growth through targeting an AR-ERG-NF-kappaB pathway in TMPRSS2/ERG fusion gene expressing prostate cancer. PloS one. 2013;8(3):e58391. Epub 2013/04/05. doi: 10.1371/journal.pone.0058391. PubMed PMID: 23554889; PubMed Central PMCID: PMC3590152.

75. Vainio P, Mpindi JP, Kohonen P, Fey V, Mirtti T, Alanen KA, et al. High-throughput transcriptomic and RNAi analysis identifies AIM1, ERGIC1, TMED3 and TPX2 as potential drug targets in prostate cancer. PloS one. 2012;7(6):e39801. Epub 2012/07/05. doi: 10.1371/journal.pone.0039801. PubMed PMID: 22761906; PubMed Central PMCID: PMC3386189.

76. Yin L, Rao P, Elson P, Wang J, Ittmann M, Heston WD. Role of TMPRSS2-ERG gene fusion in negative regulation of PSMA expression. PloS one. 2011;6(6):e21319. Epub 2011/07/07. doi: 10.1371/journal.pone.0021319. PubMed PMID: 21731703; PubMed Central PMCID: PMC3123299.

77. Zammarchi F, Boutsalis G, Cartegni L. 5' UTR control of native ERG and of Tmprss2:ERG variants activity in prostate cancer. PloS one. 2013;8(3):e49721. Epub 2013/03/09. doi: 10.1371/journal.pone.0049721. PubMed PMID: 23472063; PubMed Central PMCID: PMC3589450.

78. Clyne M. Prostate cancer: TMPRSS2:ERG--the root of the problem? Nature reviews Urology. 2013;10(5):248. Epub 2013/04/17. doi: 10.1038/nrurol.2013.80. PubMed PMID: 23588405.

79. Razzak M. Prostate cancer: Not guilty-TMPRSS2-ERG does not sensitize cells to radiation. Nature reviews Urology. 2013;10(10):556. Epub 2013/08/28. doi: 10.1038/nrurol.2013.188. PubMed PMID: 23979664.

80. Rosen P, Sesterhenn IA, Brassell SA, McLeod DG, Srivastava S, Dobi A. Clinical potential of the ERG oncoprotein in prostate cancer. Nature reviews Urology. 2012;9(3):131-7. Epub 2012/02/15. doi: 10.1038/nrurol.2012.10. PubMed PMID: 22331093.

81. Tomlins SA, Aubin SM, Siddiqui J, Lonigro RJ, Sefton-Miller L, Miick S, et al. Urine TMPRSS2:ERG fusion transcript stratifies prostate cancer risk in men with elevated serum PSA. Science translational medicine. 2011;3(94):94ra72. Epub 2011/08/05. doi: 10.1126/scitranslmed.3001970. PubMed PMID: 21813756; PubMed Central PMCID: PMC3245713.

82. Schaefer G, Mosquera JM, Ramoner R, Park K, Romanel A, Steiner E, et al. Distinct ERG rearrangement prevalence in prostate cancer: higher frequency in young age and in low PSA prostate cancer. Prostate cancer and prostatic diseases. 2013;16(2):132-8. Epub 2013/02/06. doi: 10.1038/pcan.2013.4. PubMed PMID: 23381693; PubMed Central PMCID: PMC3655380.

83. Mwamukonda K, Chen Y, Ravindranath L, Furusato B, Hu Y, Sterbis J, et al. Quantitative expression of TMPRSS2 transcript in prostate tumor cells reflects TMPRSS2-ERG fusion status. Prostate cancer and prostatic diseases. 2010;13(1):47-51. Epub 2009/07/15. doi: 10.1038/pcan.2009.28. PubMed PMID: 19597533; PubMed Central PMCID: PMC2820150.

84. Lippolis G, Edsjo A, Stenman UH, Bjartell A. A high-density tissue microarray from patients with clinically localized prostate cancer reveals ERG and TATI exclusivity in tumor cells. Prostate cancer and prostatic diseases. 2013;16(2):145-50. Epub 2013/03/06. doi: 10.1038/pcan.2013.7. PubMed PMID: 23459095; PubMed Central PMCID: PMC3655381.

85. Gumuskaya B, Gurel B, Fedor H, Tan HL, Weier CA, Hicks JL, et al. Assessing the order of critical alterations in prostate cancer development and progression by IHC: further evidence that PTEN loss occurs subsequent to ERG gene fusion. Prostate cancer and prostatic diseases. 2013;16(2):209-15. Epub 2013/04/03. doi: 10.1038/pcan.2013.8. PubMed PMID: 23545904; PubMed Central PMCID: PMC3774596.

86. Gsponer JR, Braun M, Scheble VJ, Zellweger T, Bachmann A, Perner S, et al. ERG rearrangement and protein expression in the progression to castration-resistant prostate cancer. Prostate cancer and prostatic diseases. 2014;17(2):126-31. Epub 2014/01/29. doi: 10.1038/pcan.2013.62. PubMed PMID: 24469092; PubMed Central PMCID: PMC4097053.

87. Furusato B, Tan SH, Young D, Dobi A, Sun C, Mohamed AA, et al. ERG oncoprotein expression in prostate cancer: clonal progression of ERG-positive tumor cells and potential for ERG-based stratification. Prostate cancer and prostatic diseases. 2010;13(3):228-37. Epub 2010/06/30. doi: 10.1038/pcan.2010.23. PubMed PMID: 20585344; PubMed Central PMCID: PMC3010744.

88. Braun M, Goltz D, Shaikhibrahim Z, Vogel W, Bohm D, Scheble V, et al. ERG protein expression and genomic rearrangement status in primary and metastatic prostate cancer--a comparative study of two monoclonal antibodies. Prostate cancer and prostatic diseases. 2012;15(2):165-9. Epub 2012/01/11. doi: 10.1038/pcan.2011.67. PubMed PMID: 22231490.

89. Weinmann S, Van Den Eeden SK, Haque R, Chen C, Richert-Boe K, Schwartzman J, et al. Immunohistochemical expression of ERG in the molecular epidemiology of fatal prostate cancer study. The Prostate. 2013;73(13):1371-7. Epub 2013/05/11. doi: 10.1002/pros.22684. PubMed PMID: 23661613; PubMed Central PMCID: PMC3745520.

90. Wang L, Li Y, Yang X, Yuan H, Li X, Qi M, et al. ERG-SOX4 interaction promotes epithelial-mesenchymal transition in prostate cancer cells. The Prostate. 2014;74(6):647-58. Epub 2014/01/18. doi: 10.1002/pros.22783. PubMed PMID: 24435928.

91. Udager AM, Shi Y, Tomlins SA, Alva A, Siddiqui J, Cao X, et al. Frequent discordance between ERG gene rearrangement and ERG protein expression in a rapid autopsy cohort of patients with lethal, metastatic, castration-resistant prostate cancer. Prostate. 2014. Epub 2014/07/22. doi: 10.1002/pros.22836. PubMed PMID: 25043157.

92. Tan SH, Furusato B, Fang X, He F, Mohamed AA, Griner NB, et al. Evaluation of ERG responsive proteome in prostate cancer. The Prostate. 2014;74(1):70-89. Epub 2013/10/12. doi: 10.1002/pros.22731. PubMed PMID: 24115221; PubMed Central PMCID: PMC4075339.

93. Swanson TA, Krueger SA, Galoforo S, Thibodeau BJ, Martinez AA, Wilson GD, et al. TMPRSS2/ERG fusion gene expression alters chemo- and radio-responsiveness in cell culture models of androgen independent prostate cancer. The Prostate. 2011. Epub 2011/03/12. doi: 10.1002/pros.21371. PubMed PMID: 21394739.

94. Spencer ES, Johnston RB, Gordon RR, Lucas JM, Ussakli CH, Hurtado-Coll A, et al. Prognostic value of ERG oncoprotein in prostate cancer recurrence and cause-specific mortality. The Prostate. 2013;73(9):905-12. Epub 2013/01/22. doi: 10.1002/pros.22636. PubMed PMID: 23334893; PubMed Central PMCID: PMC3677047.

95. Robert G, Jannink S, Smit F, Aalders T, Hessels D, Cremers R, et al. Rational basis for the combination of PCA3 and TMPRSS2:ERG gene fusion for prostate cancer diagnosis. The Prostate. 2013;73(2):113-20. Epub 2012/06/08. doi: 10.1002/pros.22546. PubMed PMID: 22674214.

96. Raymundo EM, Diwa MH, Lapitan MC, Plaza AB, Sevilleja JE, Srivastava S, et al. Increased association of the ERG oncoprotein expression in advanced stages of prostate cancer in Filipinos. The Prostate. 2014;74(11):1079-85. Epub 2014/06/10. doi: 10.1002/pros.22791. PubMed PMID: 24909781.

97. Nagle RB, Algotar AM, Cortez CC, Smith K, Jones C, Sathyanarayana UG, et al. ERG overexpression and PTEN status predict capsular penetration in prostate carcinoma. The Prostate. 2013;73(11):1233-40. Epub 2013/05/09. doi: 10.1002/pros.22675. PubMed PMID: 23653096; PubMed Central PMCID: PMC4038303.

98. Magi-Galluzzi C, Tsusuki T, Elson P, Simmerman K, LaFargue C, Esgueva R, et al. TMPRSS2-ERG gene fusion prevalence and class are significantly different in prostate cancer of Caucasian, African-American and Japanese patients. The Prostate. 2011;71(5):489-97. Epub 2010/09/30. doi: 10.1002/pros.21265. PubMed PMID: 20878952.

99. Grupp K, Ospina-Klinck D, Tsourlakis MC, Koop C, Wilczak W, Adam M, et al. NY-ESO-1 expression is tightly linked to TMPRSS2-ERG fusion in prostate cancer. The Prostate. 2014;74(10):1012-22. Epub 2014/05/03. doi: 10.1002/pros.22816. PubMed PMID: 24789172.

100. Grupp K, Diebel F, Sirma H, Simon R, Breitmeyer K, Steurer S, et al. SPINK1 expression is tightly linked to 6q15- and 5q21-deleted ERG-fusion negative prostate cancers but unrelated to PSA recurrence. The Prostate. 2013;73(15):1690-8. Epub 2013/07/12. doi: 10.1002/pros.22707. PubMed PMID: 23843146.

101. Dijkstra S, Leyten GHJM, Jannink SA, de Jong H, Mulders PFA, van Oort IM, et al. KLK3, PCA3, and TMPRSS2-ERG expression in the peripheral blood mononuclear cell fraction from castration-resistant prostate cancer patients and response to docetaxel treatment. The Prostate. 2014;74(12):1222-30. Epub 2014/07/22. doi: 10.1002/pros.22839. PubMed PMID: 25043536.

102. Cornu J-N, Cancel-Tassin G, Egrot C, Gaffory C, Haab F, Cussenot O. Urine TMPRSS2:ERG fusion transcript integrated with PCA3 score, genotyping, and biological features are correlated to the results of prostatic biopsies in men at risk of prostate cancer. The Prostate. 2013;73(3):242-9. Epub 2012/07/24. doi: 10.1002/pros.22563. PubMed PMID: 22821767.

103. Wang S, Kollipara RK, Srivastava N, Li R, Ravindranathan P, Hernandez E, et al. Ablation of the oncogenic transcription factor ERG by deubiquitinase inhibition in prostate cancer. Proceedings of the National Academy of Sciences of the United States of America. 2014;111(11):4251-6. Epub 2014/03/05. doi: 10.1073/pnas.1322198111. PubMed PMID: 24591637; PubMed Central PMCID: PMC3964108.

104. Regan MC, Horanyi PS, Pryor EE, Jr., Sarver JL, Cafiso DS, Bushweller JH. Structural and dynamic studies of the transcription factor ERG reveal DNA binding is allosterically autoinhibited. Proceedings of the National Academy of Sciences of the United States of America. 2013;110(33):13374-9. Epub 2013/07/31. doi: 10.1073/pnas.1301726110. PubMed PMID: 23898196; PubMed Central PMCID: PMC3746864.

105. Kruse EA, Loughran SJ, Baldwin TM, Josefsson EC, Ellis S, Watson DK, et al. Dual requirement for the ETS transcription factors Fli-1 and Erg in hematopoietic stem cells and the megakaryocyte lineage. Proceedings of the National Academy of Sciences of the United States of America. 2009;106(33):13814-9. Epub 2009/08/12. doi: 10.1073/pnas.0906556106. PubMed PMID: 19666492; PubMed Central PMCID: PMC2728977.

106. Klezovitch O, Risk M, Coleman I, Lucas JM, Null M, True LD, et al. A causal role for ERG in neoplastic transformation of prostate epithelium. Proceedings of the National Academy of Sciences of the United States of America. 2008;105(6):2105-10. Epub 2008/02/05. doi: 10.1073/pnas.0711711105. PubMed PMID: 18245377; PubMed Central PMCID: PMC2538886.

107. Tomlins SA, Laxman B, Varambally S, Cao X, Yu J, Helgeson BE, et al. Role of the TMPRSS2-ERG gene fusion in prostate cancer. Neoplasia. 2008;10(2):177-88. Epub 2008/02/20. PubMed PMID: 18283340; PubMed Central PMCID: PMC2244693.

108. Rickman DS, Chen YB, Banerjee S, Pan Y, Yu J, Vuong T, et al. ERG cooperates with androgen receptor in regulating trefoil factor 3 in prostate cancer disease progression. Neoplasia. 2010;12(12):1031-40. Epub 2010/12/21. PubMed PMID: 21170267; PubMed Central PMCID: PMC3003138.

109. Pflueger D, Rickman DS, Sboner A, Perner S, LaFargue CJ, Svensson MA, et al. N-myc downstream regulated gene 1 (NDRG1) is fused to ERG in prostate cancer. Neoplasia. 2009;11(8):804-11. Epub 2009/08/04. PubMed PMID: 19649210; PubMed Central PMCID: PMC2713587.

110. Park K, Tomlins SA, Mudaliar KM, Chiu Y-L, Esgueva R, Mehra R, et al. Antibody-based detection of ERG rearrangement-positive prostate cancer. Neoplasia. 2010;12(7):590-8. Epub 2010/07/24. PubMed PMID: 20651988; PubMed Central PMCID: PMC2907585.

111. Sreenath TL, Dobi A, Petrovics G, Srivastava S. Oncogenic activation of ERG: A predominant mechanism in prostate cancer. Journal of carcinogenesis. 2011;10:37. Epub 2012/01/27. doi: 10.4103/1477-3163.91122. PubMed PMID: 22279422; PubMed Central PMCID: PMC3263025.

112. St John J, Powell K, Conley-Lacomb MK, Chinni SR. TMPRSS2-ERG Fusion Gene Expression in Prostate Tumor Cells and Its Clinical and Biological Significance in Prostate Cancer Progression. Journal of cancer science & therapy. 2012;4(4):94-101. Epub 2012/12/25. doi: 10.4172/1948-5956.1000119. PubMed PMID: 23264855; PubMed Central PMCID: PMC3527835.

113. Rostad K, Mannelqvist M, Halvorsen OJ, Oyan AM, Bo TH, Stordrange L, et al. ERG upregulation and related ETS transcription factors in prostate cancer. International journal of oncology. 2007;30(1):19-32. Epub 2006/12/05. PubMed PMID: 17143509.

114. Mertz KD, Horcic M, Hailemariam S, D'Antonio A, Dirnhofer S, Hartmann A, et al. Heterogeneity of ERG expression in core needle biopsies of patients with early prostate cancer. Hum Pathol. 2013;44(12):2727-35. Epub 2013/10/01. doi: 10.1016/j.humpath.2013.07.019. PubMed PMID: 24074533.

115. Johnson H, Zhou M, Osunkoya AO. ERG expression in mucinous prostatic adenocarcinoma and prostatic adenocarcinoma with mucinous features: comparison with conventional prostatic adenocarcinoma. Hum Pathol. 2013;44(10):2241-6. Epub 2013/07/16. doi: 10.1016/j.humpath.2013.05.006. PubMed PMID: 23849895.

116. Demichelis F, Setlur SR, Beroukhim R, Perner S, Korbel JO, Lafargue CJ, et al. Distinct genomic aberrations associated with ERG rearranged prostate cancer. Genes, chromosomes & cancer. 2009;48(4):366-80. Epub 2009/01/22. doi: 10.1002/gcc.20647. PubMed PMID: 19156837; PubMed Central PMCID: PMC2674964.

117. Bismar TA, Dolph M, Teng L-H, Liu S, Donnelly B. ERG protein expression reflects hormonal treatment response and is associated with Gleason score and prostate cancer specific mortality. European journal of cancer. 2012;48(4):538-46. Epub 2012/02/04. doi: 10.1016/j.ejca.2012.01.001. PubMed PMID: 22300588.

118. Wang J, Cai Y, Yu W, Ren C, Spencer DM, Ittmann M. Pleiotropic biological activities of alternatively spliced TMPRSS2/ERG fusion gene transcripts. Cancer research. 2008;68(20):8516-24. Epub 2008/10/17. doi: 10.1158/0008-5472.CAN-08-1147. PubMed PMID: 18922926; PubMed Central PMCID: PMC2597580.

119. Wang J, Cai Y, Shao LJ, Siddiqui J, Palanisamy N, Li R, et al. Activation of NF-{kappa}B by TMPRSS2/ERG Fusion Isoforms through Toll-Like Receptor-4. Cancer research. 2011;71(4):1325-33. Epub 2010/12/21. doi: 10.1158/0008-5472.CAN-10-2210. PubMed PMID: 21169414; PubMed Central PMCID: PMC3041849.

120. Salek-Ardakani S, Smooha G, de Boer J, Sebire NJ, Morrow M, Rainis L, et al. ERG is a megakaryocytic oncogene. Cancer research. 2009;69(11):4665-73. Epub 2009/06/03. doi: 10.1158/0008-5472.CAN-09-0075. PubMed PMID: 19487285.

121. Hofer MD, Kuefer R, Maier C, Herkommer K, Perner S, Demichelis F, et al. Genome-wide linkage analysis of TMPRSS2-ERG fusion in familial prostate cancer. Cancer research. 2009;69(2):640-6. Epub 2009/01/17. doi: 10.1158/0008-5472.CAN-08-2008. PubMed PMID: 19147579.

122. Attard G, Swennenhuis JF, Olmos D, Reid AHM, Vickers E, A'Hern R, et al. Characterization of ERG, AR and PTEN gene status in circulating tumor cells from patients with castration-resistant prostate cancer. Cancer research. 2009;69(7):2912-8. Epub 2009/04/03. doi: 10.1158/0008-5472.CAN-08-3667. PubMed PMID: 19339269.

123. Shah RB, Tadros Y, Brummell B, Zhou M. The diagnostic use of ERG in resolving an "atypical glands suspicious for cancer" diagnosis in prostate biopsies beyond that provided by basal cell and alpha-methylacyl-CoA-racemase markers. Hum Pathol. 2013;44(5):786-94. Epub 2012/11/20. doi: 10.1016/j.humpath.2012.06.024. PubMed PMID: 23158212.

124. Minner S, Enodien M, Sirma H, Luebke AM, Krohn A, Mayer PS, et al. ERG status is unrelated to PSA recurrence in radically operated prostate cancer in the absence of antihormonal therapy. Clinical cancer research : an official journal of the American Association for Cancer Research. 2011;17(18):5878-88. Epub 2011/07/28. doi: 10.1158/1078-0432.CCR-11-1251. PubMed PMID: 21791629.

125. Magee JA, Araki T, Patil S, Ehrig T, True L, Humphrey PA, et al. Expression Profiling Reveals Hepsin Overexpression in Prostate Cancer. Cancer Research. 2001;61(15):5692-6.

126. Pace G, Pomante R, Vicentini C. Hepsin in the diagnosis of prostate cancer. Minerva urologica e nefrologica = The Italian journal of urology and nephrology. 2012;64(2):143-8. Epub 2012/05/24. PubMed PMID: 22617308.

127. Chevillet JR, Park GJ, Bedalov A, Simon JA, Vasioukhin VI. Identification and characterization of small-molecule inhibitors of hepsin. Mol Cancer Ther. 2008;7(10):3343-51. Epub 2008/10/15. doi: 10.1158/1535-7163.MCT-08-0446. PubMed PMID: 18852137; PubMed Central PMCID: PMC2659609.

128. Guo J, Li G, Tang J, Cao XB, Zhou QY, Fan ZJ, et al. HLA-A2-restricted cytotoxic T lymphocyte epitopes from human hepsin as novel targets for prostate cancer immunotherapy. Scandinavian journal of immunology. 2013;78(3):248-57. Epub 2013/06/01. doi: 10.1111/sji.12083. PubMed PMID: 23721092.

129. Li W, Wang B-E, Moran P, Lipari T, Ganesan R, Corpuz R, et al. Pegylated kunitz domain inhibitor suppresses hepsin-mediated invasive tumor growth and metastasis. Cancer research. 2009;69(21):8395-402. Epub 2009/10/22. doi: 10.1158/0008-5472.CAN-09-1995. PubMed PMID: 19843851.

130. Nandana S, Ellwood-Yen K, Sawyers C, Wills M, Weidow B, Case T, et al. Hepsin cooperates with MYC in the progression of adenocarcinoma in a prostate cancer mouse model. The Prostate. 2010;70(6):591-600. Epub 2009/11/26. doi: 10.1002/pros.21093. PubMed PMID: 19938013; PubMed Central PMCID: PMC2925264.

131. Owen KA, Qiu D, Alves J, Schumacher AM, Kilpatrick LM, Li J, et al. Pericellular activation of hepatocyte growth factor by the transmembrane serine proteases matriptase and hepsin, but not by the membrane-associated protease uPA. The Biochemical journal. 2010;426(2):219-28. Epub 2009/12/18. doi: 10.1042/BJ20091448. PubMed PMID: 20015050.

132. Tang X, Mahajan SS, Nguyen LT, Beliveau F, Leduc R, Simon JA, et al. Targeted inhibition of cell-surface serine protease Hepsin blocks prostate cancer bone metastasis. Oncotarget. 2014;5(5):1352-62. Epub 2014/03/25. PubMed PMID: 24657880; PubMed Central PMCID: PMC4012739.

133. Tripathi M, Nandana S, Yamashita H, Ganesan R, Kirchhofer D, Quaranta V. Laminin-332 is a substrate for hepsin, a protease associated with prostate cancer progression. The Journal of biological chemistry. 2008;283(45):30576-84. Epub 2008/09/12. doi: 10.1074/jbc.M802312200. PubMed PMID: 18784072; PubMed Central PMCID: PMC2576550.

134. Wittig-Blaich SM, Kacprzyk LA, Eismann T, Bewerunge-Hudler M, Kruse P, Winkler E, et al. Matrix-dependent regulation of AKT in Hepsin-overexpressing PC3 prostate cancer cells. Neoplasia. 2011;13(7):579-89. Epub 2011/07/14. PubMed PMID: 21750652; PubMed Central PMCID: PMC3132844.

135. Chen Z, Fan Z, McNeal JE, Nolley R, Caldwell MC, Mahadevappa M, et al. Hepsin and maspin are inversely expressed in laser capture microdissectioned prostate cancer. J Urology. 2003;169(4):1316-9. Epub 2003/03/12. doi: 10.1097/01.ju.0000050648.40164.0d. PubMed PMID: 12629351.

136. Herter S, Piper DE, Aaron W, Gabriele T, Cutler G, Cao P, et al. Hepatocyte growth factor is a preferred in vitro substrate for human hepsin, a membrane-anchored serine protease implicated in prostate and ovarian cancers. The Biochemical journal. 2005;390(Pt 1):125-36. Epub 2005/04/21. doi: 10.1042/BJ20041955. PubMed PMID: 15839837; PubMed Central PMCID: PMC1184568.

137. Kelly KA, Setlur SR, Ross R, Anbazhagan R, Waterman P, Rubin MA, et al. Detection of early prostate cancer using a hepsin-targeted imaging agent. Cancer research. 2008;68(7):2286-91. Epub 2008/04/03. doi: 10.1158/0008-5472.CAN-07-1349. PubMed PMID: 18381435; PubMed Central PMCID: PMC2709884.

138. Kirchhofer D, Peek M, Lipari MT, Billeci K, Fan B, Moran P. Hepsin activates pro-hepatocyte growth factor and is inhibited by hepatocyte growth factor activator inhibitor-1B (HAI-1B) and HAI-2. FEBS letters. 2005;579(9):1945-50. Epub 2005/03/29. doi: 10.1016/j.febslet.2005.01.085. PubMed PMID: 15792801.

139. Klezovitch O, Chevillet J, Mirosevich J, Roberts RL, Matusik RJ, Vasioukhin V. Hepsin promotes prostate cancer progression and metastasis. Cancer cell. 2004;6(2):185-95. Epub 2004/08/25. doi: 10.1016/j.ccr.2004.07.008. PubMed PMID: 15324701.

140. Matsuo T, Nakamura K, Takamoto N, Kodama J, Hongo A, Abrzua F, et al. Expression of the serine protease hepsin and clinical outcome of human endometrial cancer. Anticancer research. 2008;28(1A):159-64. Epub 2008/04/04. PubMed PMID: 18383840.

141. Moran P, Li W, Fan B, Vij R, Eigenbrot C, Kirchhofer D. Pro-urokinase-type plasminogen activator is a substrate for hepsin. The Journal of biological chemistry. 2006;281(41):30439-46. Epub 2006/08/16. doi: 10.1074/jbc.M605440200. PubMed PMID: 16908524.

142. Pal P, Xi H, Kaushal R, Sun G, Jin CH, Jin L, et al. Variants in the HEPSIN gene are associated with prostate cancer in men of European origin. Human genetics. 2006;120(2):187-92. Epub 2006/06/20. doi: 10.1007/s00439-006-0204-3. PubMed PMID: 16783571.

143. Srikantan V, Valladares M, Rhim JS, Moul JW, Srivastava S. HEPSIN inhibits cell growth/invasion in prostate cancer cells. Cancer research. 2002;62(23):6812-6. Epub 2002/12/04. PubMed PMID: 12460890.

144. Stephan C, Yousef GM, Scorilas A, Jung K, Jung M, Kristiansen G, et al. Hepsin is highly over expressed in and a new candidate for a prognostic indicator in prostate cancer. J Urology. 2004;171(1):187-91. Epub 2003/12/11. doi: 10.1097/01.ju.0000101622.74236.94. PubMed PMID: 14665873.

145. Vasioukhin V. Hepsin paradox reveals unexpected complexity of metastatic process. Cell cycle. 2004;3(11):1394-7. Epub 2004/11/13. PubMed PMID: 15539945.

146. Wu Q, Parry G. Hepsin and prostate cancer. Frontiers in bioscience : a journal and virtual library. 2007;12:5052-9. Epub 2007/06/16. PubMed PMID: 17569629.

147. Xuan JA, Schneider D, Toy P, Lin R, Newton A, Zhu Y, et al. Antibodies neutralizing hepsin protease activity do not impact cell growth but inhibit invasion of prostate and ovarian tumor cells in culture. Cancer research. 2006;66(7):3611-9. Epub 2006/04/06. doi: 10.1158/0008-5472.CAN-05-2983. PubMed PMID: 16585186.

148. Chambers K, Pearson J, Pellacani D, Aziz N, Guzvic M, Klein C, et al. Stromal upregulation of lateral epithelial adhesions: Gene expression analysis of signalling pathways in prostate epithelium. Journal of biomedical science. 2011;18(1):45. doi: 10.1186/1423-0127-18-45. PubMed PMID: doi:10.1186/1423-0127-18-45; PubMed Central PMCID: PMC3141633.

149. Lai Y-H, Cheng J, Cheng D, Feasel M, Beste K, Peng J, et al. SOX4 interacts with plakoglobin in a Wnt3a-dependent manner in prostate cancer cells. BMC Cell Biology. 2011;12(1):50. PubMed PMID: doi:10.1186/1471-2121-12-50.

150. Liu P, Ramachandran S, Ali Seyed M, Scharer CD, Laycock N, Dalton WB, et al. Sex-Determining Region Y Box 4 Is a Transforming Oncogene in Human Prostate Cancer Cells. Cancer Research. 2006;66(8):4011-9. doi: 10.1158/0008-5472.can-05-3055.

151. Scharer CD, McCabe CD, Ali-Seyed M, Berger MF, Bulyk ML, Moreno CS. Genome-Wide Promoter Analysis of the SOX4 Transcriptional Network in Prostate Cancer Cells. Cancer Research. 2009;69(2):709-17. doi: 10.1158/0008-5472.can-08-3415.

152. Vanaja DK, Ballman KV, Morlan BW, Cheville JC, Neumann RM, Lieber MM, et al. PDLIM4 repression by hypermethylation as a potential biomarker for prostate cancer. Clinical Cancer Research. 2006;12(4):1128-36. doi: 10.1158/1078-0432.ccr-05-2072.

153. Wang L, Li Y, Yang X, Yuan H, Li X, Qi M, et al. ERG–SOX4 interaction promotes epithelial–mesenchymal transition in prostate cancer cells. The Prostate. 2014;74(6):647-58. doi: 10.1002/pros.22783.

154. Wang L, Zhang J, Yang X, Chang YWY, Qi M, Zhou Z, et al. SOX4 is associated with poor prognosis in prostate cancer and promotes epithelial-mesenchymal transition in vitro. Prostate Cancer Prostatic Dis. 2013;16(4):301-7. doi: 10.1038/pcan.2013.25.

155. Lakshman M, Huang X, Ananthanarayanan V, Jovanovic B, Liu Y, Craft C, et al. Endoglin suppresses human prostate cancer metastasis. Clinical & Experimental Metastasis. 2011;28(1):39-53. doi: 10.1007/s10585-010-9356-6. PubMed PMID: 20981476; PubMed Central PMCID: PMC3046557.

156. Wang L, Zhang J, Yang X, Chang YW, Qi M, Zhou Z, et al. SOX4 is associated with poor prognosis in prostate cancer and promotes epithelial-mesenchymal transition in vitro. Prostate cancer and prostatic diseases. 2013;16(4):301-7. Epub 2013/08/07. doi: 10.1038/pcan.2013.25. PubMed PMID: 23917306.

157. Lai Y-H, Cheng J, Cheng D, Feasel ME, Beste KD, Peng J, et al. SOX4 interacts with plakoglobin in a Wnt3a-dependent manner in prostate cancer cells. BMC cell biology. 2011;12:50. Epub 2011/11/22. doi: 10.1186/1471-2121-12-50. PubMed PMID: 22098624; PubMed Central PMCID: PMC3227594.

158. Liu P, Ramachandran S, Ali Seyed M, Scharer CD, Laycock N, Dalton WB, et al. Sex-determining region Y box 4 is a transforming oncogene in human prostate cancer cells. Cancer research. 2006;66(8):4011-9. Epub 2006/04/19. doi: 10.1158/0008-5472.CAN-05-3055. PubMed PMID: 16618720.

159. Moreno CS. The Sex-determining region Y-box 4 and homeobox C6 transcriptional networks in prostate cancer progression: crosstalk with the Wnt, Notch, and PI3K pathways. The American journal of pathology. 2010;176(2):518-27. Epub 2009/12/19. doi: 10.2353/ajpath.2010.090657. PubMed PMID: 20019190; PubMed Central PMCID: PMC2808058.

160. Scharer CD, McCabe CD, Ali-Seyed M, Berger MF, Bulyk ML, Moreno CS. Genome-wide promoter analysis of the SOX4 transcriptional network in prostate cancer cells. Cancer research. 2009;69(2):709-17. Epub 2009/01/17. doi: 10.1158/0008-5472.CAN-08-3415. PubMed PMID: 19147588; PubMed Central PMCID: PMC2629396.

161. Haram KM, Peltier HJ, Lu B, Bhasin M, Otu HH, Choy B, et al. Gene expression profile of mouse prostate tumors reveals dysregulations in major biological processes and identifies potential murine targets for preclinical development of human prostate cancer therapy. The Prostate. 2008;68(14):1517-30. Epub 2008/08/01. doi: 10.1002/pros.20803. PubMed PMID: 18668517.

162. Vanaja DK, Ballman KV, Morlan BW, Cheville JC, Neumann RM, Lieber MM, et al. PDLIM4 repression by hypermethylation as a potential biomarker for prostate cancer. Clinical cancer research : an official journal of the American Association for Cancer Research. 2006;12(4):1128-36. Epub 2006/02/21. doi: 10.1158/1078-0432.CCR-05-2072. PubMed PMID: 16489065.

163. Klee EW, Bondar OP, Goodmanson MK, Dyer RB, Erdogan S, Bergstralh EJ, et al. Candidate Serum Biomarkers for Prostate Adenocarcinoma Identified by mRNA Differences in Prostate Tissue and Verified with Protein Measurements in Tissue and Blood. Clinical chemistry. 2012;58(3):599-609. doi: 10.1373/clinchem.2011.171637. PubMed PMID: 22247499; PubMed Central PMCID: PMC3951013.

164. Yamamoto-Ishikawa K SH, Nezu M, Kamiya N, Imamoto T, et al. The isolation and identification of apolipoprotein C-I in hormone-refractory prostate cancer using surface-enhanced laser desorption/ionization time-of-flight mass spectrometry. . Asian Journal of Andrology. 2009;11(3):299–307.

165. Engwegen JYMN, Helgason HH, Cats A, Harris N, Bonfrer JMG, Schellens JHM, et al. Identification of serum proteins discriminating colorectal cancer patients and healthy controls using surface-enhanced laser desorption ionisation-time of flight mass spectrometry. World journal of gastroenterology : WJG. 2006;12(10):1536-44. Epub 2006/03/30. PubMed PMID: 16570345; PubMed Central PMCID: PMC4124285.

166. Kwan PS, Lau CC, Chiu YT, Man C, Liu J, Tang KD, et al. Daxx regulates mitotic progression and prostate cancer predisposition. Carcinogenesis. 2013;34(4):750-9. doi: 10.1093/carcin/bgs391. PubMed PMID: 23239745.

167. Lin D-Y, Fang H-I, Ma A-H, Huang Y-S, Pu Y-S, Jenster G, et al. Negative Modulation of Androgen Receptor Transcriptional Activity by Daxx. Molecular and Cellular Biology. 2004;24(24):10529-41. doi: 10.1128/mcb.24.24.10529-10541.2004.

168. Song JJ, Lee YJ. Role of the ASK1-SEK1-JNK1-HIPK1 Signal in Daxx Trafficking and ASK1 Oligomerization. Journal of Biological Chemistry. 2003;278(47):47245-52. doi: 10.1074/jbc.M213201200.

169. Tsourlakis MC, Schoop M, Plass C, Huland H, Graefen M, Steuber T, et al. Overexpression of the chromatin remodeler death-domain–associated protein in prostate cancer is an independent predictor of early prostate-specific antigen recurrence. Hum Pathol. 2013;44(9):1789-96. doi: 10.1016/j.humpath.2013.01.022.

170. Waghray A, Schober M, Feroze F, Yao F, Virgin J, Chen YQ. Identification of Differentially Expressed Genes by Serial Analysis of Gene Expression in Human Prostate Cancer. Cancer Research. 2001;61(10):4283-6.

171. Jia L, Yu W, Wang P, Li J, Sanders BG, Kline K. Critical roles for JNK, c-Jun, and Fas/FasL-Signaling in vitamin E analog-induced apoptosis in human prostate cancer cells. The Prostate. 2008;68(4):427-41. doi: 10.1002/pros.20716.

172. Song MS, Salmena L, Carracedo A, Egia A, Lo-Coco F, Teruya-Feldstein J, et al. The deubiquitinylation and localization of PTEN are regulated by a HAUSP-PML network. Nature. 2008;455(7214):813-7. doi: <http://www.nature.com/nature/journal/v455/n7214/suppinfo/nature07290_S1.html>.

173. Bernkopf DB, Williams ED. Potential role of EPB41L3 (Protein 4.1B/Dal-1) as a target for treatment of advanced prostate cancer. Expert opinion on therapeutic targets. 2008;12(7):845-53. doi: 10.1517/14728222.12.7.845. PubMed PMID: 18554153.

174. Schulz W, Alexa A, Jung V, Hader C, Hoffmann M, Yamanaka M, et al. Factor interaction analysis for chromosome 8 and DNA methylation alterations highlights innate immune response suppression and cytoskeletal changes in prostate cancer. Molecular Cancer. 2007;6(1):14. PubMed PMID: doi:10.1186/1476-4598-6-14.

175. Schulz W, Ingenwerth M, Djuidje C, Hader C, Rahnenführer J, Engers R. Changes in cortical cytoskeletal and extracellular matrix gene expression in prostate cancer are related to oncogenic ERG deregulation. BMC cancer. 2010;10(1):1-9. doi: 10.1186/1471-2407-10-505.

176. Engl T, Relja B, Blumenberg C, Müller I, Ringel EM, Beecken W-D, et al. Prostate tumor CXC-chemokine profile correlates with cell adhesion to endothelium and extracellular matrix. Life sciences. 2006;78(16):1784-93. doi: <http://dx.doi.org/10.1016/j.lfs.2005.08.019>. PubMed PMID: 16263140.

177. König JE, Senge T, Allhoff EP, König W. Analysis of the inflammatory network in benign prostate hyperplasia and prostate cancer. The Prostate. 2004;58(2):121-9. doi: 10.1002/pros.10317. PubMed PMID: 14716737.

178. Nagpal ML, Chen Y, Lin T. Effects of overexpression of CXCL10 (cytokine-responsive gene-2) on MA-10 mouse Leydig tumor cell steroidogenesis and proliferation. The Journal of endocrinology. 2004;183(3):585-94. doi: 10.1677/joe.1.05795. PubMed PMID: 15590984.

179. Nagpal ML, Davis J, Lin T. Overexpression of CXCL10 in human prostate LNCaP cells activates its receptor (CXCR3) expression and inhibits cell proliferation. Biochimica et biophysica acta. 2006;1762(9):811-8. doi: 10.1016/j.bbadis.2006.06.017. PubMed PMID: 16934957.

180. Shen H, Schuster R, Lu B, Waltz SE, Lentsch AB. Critical and opposing roles of the chemokine receptors CXCR2 and CXCR3 in prostate tumor growth. The Prostate. 2006;66(16):1721-8. doi: 10.1002/pros.20476.

181. Wedel S, Raditchev, I., Jones, J., Juengel, E., Engl, T., Jonas, D., & Blaheta, R. CXC chemokine mRNA expression as a potential diagnostic tool in prostate cancer. Molecular Medicine Reports. 2008;1(2):257-62.

182. Wu Q, Dhir R, Wells A. Altered CXCR3 isoform expression regulates prostate cancer cell migration and invasion. Molecular Cancer. 2012;11(1):3. PubMed PMID: doi:10.1186/1476-4598-11-3.

183. Chen C-L, Mahalingam D, Osmulski P, Jadhav RR, Wang C-M, Leach RJ, et al. Single-cell analysis of circulating tumor cells identifies cumulative expression patterns of EMT-related genes in metastatic prostate cancer. The Prostate. 2013;73(8):813-26. Epub 2013/01/03. doi: 10.1002/pros.22625. PubMed PMID: 23280481.

184. Liu Y, Song H, Pan J, Zhao J. Comprehensive gene expression analysis reveals multiple signal pathways associated with prostate cancer. J Appl Genetics. 2014;55(1):117-24. doi: 10.1007/s13353-013-0174-9.

185. Reinertsen T, Halgunset J, Viset T, Flatberg A, Haugsmoen LL, Skogseth H. Gene expressional changes in prostate fibroblasts from cancerous tissue. APMIS. 2012;120(7):558-71. doi: 10.1111/j.1600-0463.2011.02865.x.

186. Schmidt M, Asirvatham A, Chaudhary J. Inhibitor of differentiation 1 (Id1) promotes cell survival and proliferation of prostate epithelial cells. Cell Mol Biol Lett. 2010;15(2):272-95. doi: 10.2478/s11658-010-0007-3.

187. Varisli L. Identification of New Genes Downregulated in Prostate Cancer and Investigation of Their Effects on Prognosis Genetic Testing and Molecular Biomarkers. 2013;17(7):562-6. doi: july 2.

188. Assinder SJ, Au E, Dong Q, Winnick C. A novel splice variant of the β-tropomyosin (TPM2) gene in prostate cancer. Molecular Carcinogenesis. 2010;49(6):525-31. doi: 10.1002/mc.20626.

189. Oji Y, Tatsumi N, Fukuda M, Nakatsuka S-I, Aoyagi S, Hirata E, et al. The translation elongation factor eEF2 is a novel tumorassociated antigen overexpressed in various types of cancers. International journal of oncology. 2014;44(5):1461-9. Epub 2014/03/05. doi: 10.3892/ijo.2014.2318. PubMed PMID: 24589652; PubMed Central PMCID: PMC4027928.

190. Wullner U, Neef I, Eller A, Kleines M, Tur MK, Barth S. Cell-specific induction of apoptosis by rationally designed bivalent aptamer-siRNA transcripts silencing eukaryotic elongation factor 2. Current cancer drug targets. 2008;8(7):554-65. doi: 10.2174/156800908786241078. PubMed PMID: 18991566.

191. Li L, Chen S-h, Yu C-h, Li Y-m, Wang S-q. Identification of Hepatocellular-Carcinoma-Associated Antigens and Autoantibodies by Serological Proteome Analysis Combined with Protein Microarray. Journal of proteome research. 2007;7(2):611-20. doi: 10.1021/pr070525r. PubMed PMID: 18161940.

192. Hong M-G, Karlsson R, Magnusson PKE, Lewis MR, Isaacs W, Zheng LS, et al. A Genome-Wide Assessment of Variability in Human Serum Metabolism. Human Mutation. 2013;34(3):515-24. doi: 10.1002/humu.22267.

193. Hurst R, Elliott RM, Goldson AJ, Fairweather-Tait SJ. Se-methylselenocysteine alters collagen gene and protein expression in human prostate cells. Cancer letters. 2008;269(1):117-26. doi: 10.1016/j.canlet.2008.04.025. PubMed PMID: 18514395.

194. Altintas DM, Allioli N, Decaussin M, de Bernard S, Ruffion A, Samarut J, et al. Differentially Expressed Androgen-Regulated Genes in Androgen-Sensitive Tissues Reveal Potential Biomarkers of Early Prostate Cancer. PloS one. 2013;8(6):e66278. doi: 10.1371/journal.pone.0066278. PubMed PMID: 23840433; PubMed Central PMCID: PMC3696068.

195. Chen Z, Gulzar ZG, St. Hill CA, Walcheck B, Brooks JD. Increased expression of GCNT1 is associated with altered O-glycosylation of PSA, PAP, and MUC1 in human prostate cancers. The Prostate. 2014;74(10):1059-67. doi: 10.1002/pros.22826. PubMed PMID: 24854630.

196. Petrosyan A, Holzapfel MS, Muirhead DE, Cheng P-W. Restoration of Compact Golgi Morphology in Advanced Prostate Cancer Enhances Susceptibility to Galectin-1-induced Apoptosis by Modifying Mucin O-glycan Synthesis. Molecular cancer research : MCR. 2014;12(12):1704-16. doi: 10.1158/1541-7786.mcr-14-0291-t. PubMed PMID: 25086069; PubMed Central PMCID: PMC4272641.

197. Dong Y, Zhang H, Gao AC, Marshall JR, Ip Ca, author = Rieger, Kerri E., Hong W-J, et al. Androgen receptor signaling intensity is a key factor in determining the sensitivity of prostate cancer cells to selenium inhibition of growth and cancer-specific biomarkers. Mol Cancer Ther. 2005;4(7):1047-55.

198. Huang D, Casale GP, Tian J, Lele SM, Pisarev VM, Simpson MA, et al. Udp-glucose dehydrogenase as a novel field-specific candidate biomarker of prostate cancer. International journal of cancer Journal international du cancer. 2010;126(2):315-27. Epub 2009/08/14. doi: 10.1002/ijc.24820. PubMed PMID: 19676054; PubMed Central PMCID: PMC2794918.

199. Hyde AS, Thelen AM, Barycki JJ, Simpson MA. UDP-glucose dehydrogenase activity and optimal downstream cellular function require dynamic reorganization at the dimer-dimer subunit interfaces. The Journal of biological chemistry. 2013;288(49):35049-57. Epub 2013/10/23. doi: 10.1074/jbc.M113.519090. PubMed PMID: 24145036; PubMed Central PMCID: PMC3853257.

200. Wei Q, Galbenus R, Raza A, Cerny RL, Simpson MA. Androgen-stimulated UDP-glucose dehydrogenase expression limits prostate androgen availability without impacting hyaluronan levels. Cancer research. 2009;69(6):2332-9. Epub 2009/02/27. doi: 10.1158/0008-5472.CAN-08-3083. PubMed PMID: 19244115; PubMed Central PMCID: PMC2657818.

201. Bar-Shira A, Pinthus JH, Rozovsky U, Goldstein M, Sellers WR, Yaron Y, et al. Multiple genes in human 20q13 chromosomal region are involved in an advanced prostate cancer xenograft. Cancer research. 2002;62(23):6803-7. Epub 2002/12/04. PubMed PMID: 12460888.

202. Szczyrba J, Nolte E, Hart M, Doll C, Wach S, Taubert H, et al. Identification of ZNF217, hnRNP-K, VEGF-A and IPO7 as targets for microRNAs that are downregulated in prostate carcinoma. International journal of cancer Journal international du cancer. 2013;132(4):775-84. Epub 2012/07/21. doi: 10.1002/ijc.27731. PubMed PMID: 22815235.

203. Thorsen K, Schepeler T, Oster B, Rasmussen M, Vang S, Wang K, et al. Tumor-specific usage of alternative transcription start sites in colorectal cancer identified by genome-wide exon array analysis. BMC Genomics. 2011;12(1):505. PubMed PMID: doi:10.1186/1471-2164-12-505.

204. Kozakowski N, Hartmann C, Klingler H, Susani M, Mazal P, Scharrer A, et al. Immunohistochemical expression of PDGFR, VEGF-C, and proteins of the mToR pathway before and after androgen deprivation therapy in prostate carcinoma: significant decrease after treatment. Targ Oncol. 2013:1-8. doi: 10.1007/s11523-013-0298-1.

205. Karacosta LG, Foster BA, Azabdaftari G, Feliciano DM, Edelman AM. A Regulatory Feedback Loop Between Ca2+/Calmodulin-dependent Protein Kinase Kinase 2 (CaMKK2) and the Androgen Receptor in Prostate Cancer Progression. Journal of Biological Chemistry. 2012;287(29):24832-43. doi: 10.1074/jbc.M112.370783.

206. Racioppi L. CaMKK2: a novel target for shaping the androgen-regulated tumor ecosystem. Trends in Molecular Medicine. 2013;19(2):83-8. doi: <http://dx.doi.org/10.1016/j.molmed.2012.12.004>.

207. Racioppi L, Means AR. Calcium/Calmodulin-dependent Protein Kinase Kinase 2: Roles in Signaling and Pathophysiology. Journal of Biological Chemistry. 2012;287(38):31658-65. doi: 10.1074/jbc.R112.356485.

208. Shima T, Mizokami A, Miyagi T, Kawai K, Izumi K, Kumaki M, et al. Down-regulation of calcium/calmodulin-dependent protein kinase kinase 2 by androgen deprivation induces castration-resistant prostate cancer. The Prostate. 2012;72(16):1789-801. doi: 10.1002/pros.22533.

209. Jariwala U, Prescott J, Jia L, Barski A, Pregizer S, Cogan J, et al. Identification of novel androgen receptor target genes in prostate cancer. Molecular Cancer. 2007;6(1):39. PubMed PMID: doi:10.1186/1476-4598-6-39.

210. Leiblich A, Cross SS, Catto JW, Phillips JT, Leung HY, Hamdy FC, et al. Lactate dehydrogenase-B is silenced by promoter hypermethylation in human prostate cancer. Oncogene. 2006;25(20):2953-60. Epub 2006/03/21. doi: 10.1038/sj.onc.1209262. PubMed PMID: 16547507.

211. Cui Y, Nadiminty N, Liu C, Lou W, Schwartz CT, Gao AC. Upregulation of glucose metabolism by NF-κB2/p52 mediates enzalutamide resistance in castration-resistant prostate cancer cells. Endocrine-Related Cancer. 2014;21(3):435-42. doi: 10.1530/erc-14-0107.

212. Szczyrba J, Loprich E, Wach S, Jung V, Unteregger G, Barth S, et al. The microRNA profile of prostate carcinoma obtained by deep sequencing. Molecular cancer research : MCR. 2010;8(4):529-38. Epub 2010/04/01. doi: 10.1158/1541-7786.MCR-09-0443. PubMed PMID: 20353999.

213. Wei S, Dunn TA, Isaacs WB, De Marzo AM, Luo J. GOLPH2 and MYO6: putative prostate cancer markers localized to the Golgi apparatus. The Prostate. 2008;68(13):1387-95. Epub 2008/06/11. doi: 10.1002/pros.20806. PubMed PMID: 18543251; PubMed Central PMCID: PMC4124602.

214. Qin Q, Xu Y, He T, Qin C, Xu J. Normal and disease-related biological functions of Twist1 and underlying molecular mechanisms. Cell Res. 2012;22(1):90-106.

215. Suhovskih AV, Tsidulko AY, Kutsenko OS, Kovner AV, Aidagulova SV, Ernberg I, et al. Transcriptional activity of heparan sulfate biosynthetic machinery is specifically impaired in benign prostate hyperplasia and prostate cancer. Frontiers in Oncology. 2014;4. doi: 10.3389/fonc.2014.00079.

216. Ma W, Diep K, Fritsche HA, Shore N, Albitar M. Diagnostic and prognostic scoring system for prostate cancer using urine and plasma biomarkers. Genetic testing and molecular biomarkers. 2014;18(3):156-63. Epub 2014/02/12. doi: 10.1089/gtmb.2013.0424. PubMed PMID: 24512523.

217. Tavassoli P, Wafa LA, Cheng H, Zoubeidi A, Fazli L, Gleave M, et al. TAF1 Differentially Enhances Androgen Receptor Transcriptional Activity via Its N-Terminal Kinase and Ubiquitin-Activating and -Conjugating Domains. Molecular Endocrinology. 2010;24(4):696-708. doi: 10.1210/me.2009-0229. PubMed PMID: 20181722.

218. Cheng W-S, Kraaij R, Nilsson B, van der Weel L, de Ridder CMA, Totterman TH, et al. A novel TARP-promoter-based adenovirus against hormone-dependent and hormone-refractory prostate cancer. Molecular therapy : the journal of the American Society of Gene Therapy. 2004;10(2):355-64. Epub 2004/08/06. doi: 10.1016/j.ymthe.2004.05.022. PubMed PMID: 15294182.

219. Epel M, Carmi I, Soueid-Baumgarten S, Oh SK, Bera T, Pastan I, et al. Targeting TARP, a novel breast and prostate tumor-associated antigen, with T cell receptor-like human recombinant antibodies. European journal of immunology. 2008;38(6):1706-20. Epub 2008/05/01. doi: 10.1002/eji.200737524. PubMed PMID: 18446790; PubMed Central PMCID: PMC2682370.

220. Fritzsche FR, Stephan C, Gerhardt J, Lein M, Hofmann I, Jung K, et al. Diagnostic and prognostic value of T-cell receptor gamma alternative reading frame protein (TARP) expression in prostate cancer. Histology and histopathology. 2010;25(6):733-9. Epub 2010/04/09. PubMed PMID: 20376779.

221. Hillerdal V, Nilsson B, Carlsson B, Eriksson F, Essand M. T cells engineered with a T cell receptor against the prostate antigen TARP specifically kill HLA-A2+ prostate and breast cancer cells. Proceedings of the National Academy of Sciences of the United States of America. 2012;109(39):15877-81. Epub 2012/09/29. doi: 10.1073/pnas.1209042109. PubMed PMID: 23019373; PubMed Central PMCID: PMC3465394.

222. Muthana M, Giannoudis A, Scott SD, Fang H-Y, Coffelt SB, Morrow FJ, et al. Use of macrophages to target therapeutic adenovirus to human prostate tumors. Cancer research. 2011;71(5):1805-15. Epub 2011/01/15. doi: 10.1158/0008-5472.CAN-10-2349. PubMed PMID: 21233334.

223. Nguyen MC, Tu GH, Koprivnikar KE, Gonzalez-Edick M, Jooss KU, Harding TC. Antibody responses to galectin-8, TARP and TRAP1 in prostate cancer patients treated with a GM-CSF-secreting cellular immunotherapy. Cancer immunology, immunotherapy : CII. 2010;59(9):1313-23. Epub 2010/05/26. doi: 10.1007/s00262-010-0858-5. PubMed PMID: 20499060.

224. Wolfgang CD, Essand M, Lee B, Pastan I. T-cell receptor gamma chain alternate reading frame protein (TARP) expression in prostate cancer cells leads to an increased growth rate and induction of caveolins and amphiregulin. Cancer research. 2001;61(22):8122-6. Epub 2001/11/24. PubMed PMID: 11719440.
